# α1-Antitrypsin attenuates microglial NLR family pyrin domain containing 3 inflammasome activation via cannabinoid receptor 2 signaling to ameliorate α-synucleinopathy-related behavioral deficits

**DOI:** 10.1186/s43556-026-00583-5

**Published:** 2026-09-20

**Authors:** Linjuan Feng, Hsuan Lo, Weipin Weng, Jiahao Zheng, Yixin Sun, Wei Lin, Xiaochun Chen, Yanping Wang, Xiaodong Pan

**Affiliations:** 1 Department of Neurology, Fujian Medical University Union Hospital, 29 Xinquan Road, Fuzhou, 350001 China; 2 Department of Geriatrics, Fujian Institute of Geriatrics, Fujian Key Laboratory of Vascular Aging, Fujian Clinical Research Center for Senile Vascular Aging and Brain Aging, Fujian Medical University Union Hospital, 29 Xinquan Road, Fuzhou, 350001 China; 3 Department of Applied Biology and Chemical Technology, The Hong Kong Polytechnic University, Hung Hom, Hong Kong, China; 4 Department of Neurology, Center for Cognitive Neurology, Shengli Clinical Medical College of Fujian Medical University, Center for Neurological Disorders, Fuzhou University Affiliated Provincial Hospital, No. 134 East Street, Gulou District, Fuzhou, 350001 China; 5 Fujian Key Laboratory of Molecular Neurology and Institute of Neuroscience, Fujian Medical University, 88 Jiaotong Road, Fuzhou, 350001 China; 6 Department of Endocrinology, Fujian Medical University Union Hospital, Fuzhou, 350001 China

**Keywords:** α1-Antitrypsin (AAT), Cannabinoid receptor 2 (CB2R), α-Synucleinopathies, Microglial NLRP3 inflammasome, Nucleus accumbens (NAc), Dopamine receptor type 2-expressing medium spiny neurons (D2-MSNs)

## Abstract

α-Synucleinopathies are marked by persistent neuroinflammation and disabling non-motor symptoms involving nucleus accumbens (NAc) dysfunction, yet the neuroimmune mechanisms linking microglial activation to accumbal synaptic pathology remain poorly understood. Here, we identify α1-antitrypsin (AAT) as a previously unrecognized modulator of cannabinoid receptor 2 (CB2R)-associated signaling in α-syn pathology. An acute transcriptomic screen revealed prominent induction of the *Serpina1* gene family, while primary-microglial and chronic AAV–α-syn experiments showed that *Cnr2* deficiency amplified *Serpina1*/AAT responses, consistent with an insufficient compensatory reaction to persistent inflammation. Molecular docking, reciprocal co-immunoprecipitation, and surface plasmon resonance provided complementary evidence supporting an AAT–CB2R association under the respective assay conditions. Functionally, AAT reduced the α-syn-associated elevation of intracellular cAMP in an AM630-sensitive manner and attenuated ATP-evoked Ca^2^⁺ responses, calpain-1 activity, GSK-3β N-terminal cleavage, and NLRP3/caspase-1-related cytokine production; these effects were substantially diminished in *Cnr2*-deficient microglia. Calpeptin reproduced key molecular effects, implicating calpain-related proteolysis in this neuroimmune response. In vivo, intracerebroventricular AAT preserved NAc synaptic ultrastructure, ameliorated excitatory synaptic abnormalities in dopamine D2 receptor-expressing medium spiny neurons, and improved fear-memory retrieval, spontaneous alternation, and anxiety-like behavior, without affecting novel object recognition or motor performance. Several effects were attenuated under *Cnr2*-deficient conditions, although selected electrophysiological responses persisted. Our findings identify AAT–CB2R-linked signaling as a modulator of microglial inflammatory homeostasis and support the therapeutic potential of AAT for NLRP3-associated neuroinflammation and non-motor dysfunction in α-synucleinopathies.

**Supplementary Information:**

The online version contains supplementary material available at https://doi.org/10.1186/s43556-026-00583-5.

## Introduction

α-Synucleinopathies comprise a group of neurodegenerative disorders characterized by the abnormal aggregation of α-synuclein within neuronal and/or glial inclusions [1]. Although motor dysfunction is a prominent clinical feature of Parkinson’s disease (PD), affective and cognitive disturbances are common non-motor manifestations and may emerge early in the disease course [2]. The nucleus accumbens (NAc), a key component of mesolimbic circuitry, has been implicated in affective and motivational processing [3–5]. Experimental manipulation of the parafascicular thalamus–NAc pathway modulates depression-like behavior in PD mice [3], whereas an amygdala–NAc circuit has been shown to encode negative valence [4]. Consistent with these experimental findings, altered NAc functional connectivity has been associated with the subsequent development of apathy in patients with PD [5]. Together, these observations identify the NAc as a relevant circuit-level substrate for selected non-motor manifestations of PD.

Within this circuitry, local neuroimmune interactions may provide an important link between α-syn pathology and synaptic dysfunction. Microglia can shape NAc circuitry through complement-dependent phagocytic remodeling [6]. Under pathological conditions, aggregated α-syn can activate innate immune pathways, including NOD-, LRR- and pyrin domain-containing protein 3 (NLRP3) inflammasome signaling [7]. NLRP3 inflammasome assembly activates caspase-1 and promotes the maturation of interleukin-1β (IL-1β) and interleukin-18 (IL-18), thereby amplifying neuroinflammatory responses and contributing to neuronal injury [8]. However, the endogenous mechanisms that constrain α-syn-associated microglial inflammatory responses within the NAc remain incompletely understood.

The cannabinoid type 2 receptor (CB2R) has emerged as a potential immunomodulatory target in neuroinflammatory disorders [9]. Although CB2R expression is relatively low in the healthy central nervous system, it can increase in glial and immune-cell populations under inflammatory conditions [10]. Pharmacological activation of CB2R has been associated with attenuation of NLRP3-related signaling and reduced production of pro-inflammatory mediators [11]. CB2R signaling in striatal and related neural circuits has also been linked to the regulation of behavioral responses [10, 12]. Consistent with these observations, our previous study showed that constitutive *Cnr2* deficiency enhanced α-syn-associated microglial activation and synaptic engulfment in the NAc [13], suggesting that CB2R-associated signaling contributes to the neuroimmune regulation of this circuitry.

α1-Antitrypsin (AAT), encoded by *SERPINA1* in humans and by the *Serpina1* gene family in mice, is a multifunctional acute-phase serpin with protease-inhibitory, immunoregulatory, and tissue-protective properties [14]. Alterations in AAT abundance and molecular structure have been reported in Alzheimer’s disease and parkinsonian disorders [15, 16], suggesting a potential association with neurodegenerative disease processes. AAT has also been shown to attenuate amyloid-β-induced microglial inflammatory responses and cytotoxicity [17]. However, its functional relevance and signaling mechanisms in α-synucleinopathies remain poorly defined. Given the established role of CB2R in regulating microglial inflammatory responses and NLRP3-related signaling, we hypothesized that AAT may influence CB2R-associated signaling and thereby attenuate α-syn-induced microglial inflammation and synaptic dysfunction.

To address this hypothesis, we combined constitutive *Cnr2*-knockout mice with NAc-targeted α-syn overexpression, primary microglial cultures, biochemical and biophysical analyses, electrophysiological recordings, and behavioral assessments. We examined whether *Cnr2* deficiency altered endogenous *Serpina1*/AAT responses and whether exogenous AAT modulated Ca^2^⁺/calpain/GSK-3β/NLRP3-related signaling. We further evaluated excitatory synaptic transmission in NAc dopamine receptor type 2-expressing medium spiny neurons (D2-MSNs), synaptic and organelle ultrastructure, and selected affective and cognitive outcomes. Our findings show that *Cnr2* deficiency enhances α-syn-associated *Serpina1*/AAT responses, whereas exogenous AAT attenuates microglial inflammatory signaling, ameliorates selected D2-MSN synaptic abnormalities, and improves behavioral deficits. Collectively, these results support an AAT–CB2R-associated neuroimmune framework in which CB2R signaling contributes substantially to the anti-inflammatory, synaptic, and behavioral effects of AAT, while complementary CB2R-independent mechanisms may also be involved.

## Results

### *Cnr2* deficiency alters the early NAc transcriptional response to α-syn, with prominent *Serpina1* family upregulation

**Fig. 1:**
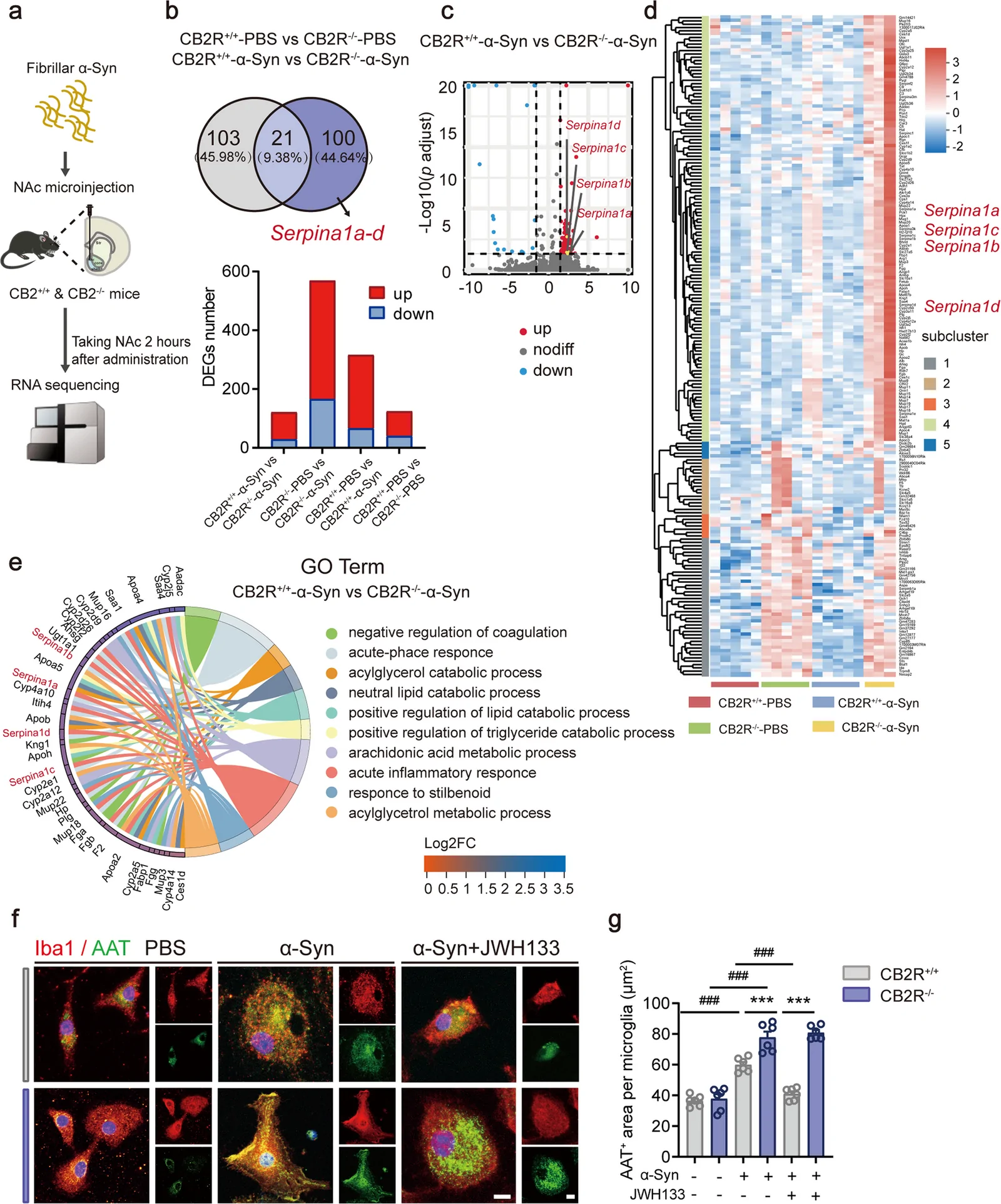
CB2R deficiency upregulates *Serpina1* gene and AAT expression following α-syn induction. **a** Schema of RNA sequencing in the NAc affected by fibrillar α-syn in CB2R^+/+^ and CB2R^−/−^ mice. **b** Venn diagram of the intersection and specific distribution of DEGs among CB2R^+/+^ and CB2R^−/−^ mice treated with PBS and α-syn. **c** Volcano diagram of the DEGs between CB2R^+/+^ and CB2R^−/−^ mice treated with α-syn. **d** Heatmap depicting the significant upregulation (red) and downregulation (blue) of DEGs from the NAc across four groups. *n* = 3 or 5 per group. **e** GO enrichment chord diagram of the DEGs corresponding to the top 10 significantly-enriched GO terms. **f** IF of Iba1 (red) and AAT (green) in primary microglia isolated from CB2R^+/+^ and CB2R^−/−^ mice treated with PBS, α-syn, α-syn + JWH133. scale bar: 10 μm (*n* = 6). **g** Quantitative analysis of the AAT-positive area per microglia. Data are presented as the mean ± SEM and assessed by two-way ANOVA with Tukey's multiple comparisons test. ^#^*p* < 0.05, ^##^*p* < 0.01, ^###^*p* < 0.001, between microglia treated with PBS, α-Syn and α-Syn + JWH133; *p < 0.05, **p < 0.01, ***p < 0.001 for genotype comparisons under the same treatment condition. NAc, Nucleus accumbens; α-syn, α-synuclein; AAT, alpha-1-antitrypsin; CB2R (*Cnr2*), Cannabinoid receptor 2; GO, Gene ontology; IF, immunofluorescence

To characterize early genotype-associated transcriptional responses to α-syn, wild-type (CB2R^+/+^) and constitutive *Cnr2*-knockout (CB2R^−/−^) mice received fibrillar α-syn or PBS, and NAc tissue was collected 2 h later for RNA sequencing (Fig. 1a). Consistent with our previous findings [13], α-syn exposure increased CB2R immunoreactivity in Iba1-positive cells in CB2R^+/+^ mice, whereas CB2R signal was not detected in CB2R^−/−^ mice (Fig. S1a-b), confirming the loss of receptor expression in the knockout animals. Principal component analysis revealed separation among the treatment–genotype groups (Fig. S2a), indicating that both α-syn exposure and *Cnr2* genotype influenced the early transcriptional response in the NAc. Among approximately 14,000 detected genes, 121 differentially expressed genes were identified by DESeq2 in α-syn-treated CB2R^−/−^ mice relative to α-syn-treated CB2R^+/+^ littermates using the predefined thresholds of |log2FC|≥ 1 and false discovery rate (FDR) ≤ 0.05 (Fig. 1b, c, Fig. S2b). Members of the *Serpina1* gene family, including *Serpina1a*, *Serpina1b*, *Serpina1c*, and *Serpina1d*, were among the most prominently upregulated transcripts in the CB2R^−/−^ group (Fig. 1c, d, Fig. S2c-d). Gene Ontology enrichment analysis of the genotype-associated differentially expressed genes highlighted acute inflammatory response-related terms (Fig. 1e). Kyoto Encyclopedia of Genes and Genomes pathway analysis further identified alterations in peroxisome proliferator-activated receptor signaling, lipid and cholesterol metabolism, and inflammatory pathways (Fig. S2e). Expression-network analysis placed the *Serpina1* transcripts within related inflammatory and metabolic modules (Fig. S2f).

Building on the early-response RNA-seq findings, we next examined whether pharmacological CB2R activation was associated with changes in microglial AAT immunoreactivity. Primary microglia isolated from CB2R^+/+^ and CB2R^−/−^ mice were exposed to PBS, α-syn, or α-syn together with the CB2R agonist JWH133. In CB2R^+/+^ microglia, JWH133 significantly attenuated the α-syn-associated increase in AAT immunoreactivity, whereas no comparable reduction was detected in CB2R^−/−^ cells (Fig. 1f, g). Together with the prominent upregulation of *Serpina1* family transcripts in the acute NAc transcriptome, these findings identify *Serpina1*/AAT as a CB2R-associated component of the early response to α-syn and indicate that pharmacological CB2R activation can modulate microglial AAT abundance under the conditions examined. The 2-h RNA-seq dataset was therefore used to nominate *Serpina1*/AAT for subsequent mechanistic investigation, rather than to infer direct temporal progression to the chronic AAV–α-syn phenotype.

### Complementary analyses support an AAT–CB2R association and reveal enhanced *Serpina1*/AAT responses in *Cnr2*-deficient α-syn pathology

**Fig. 2:**
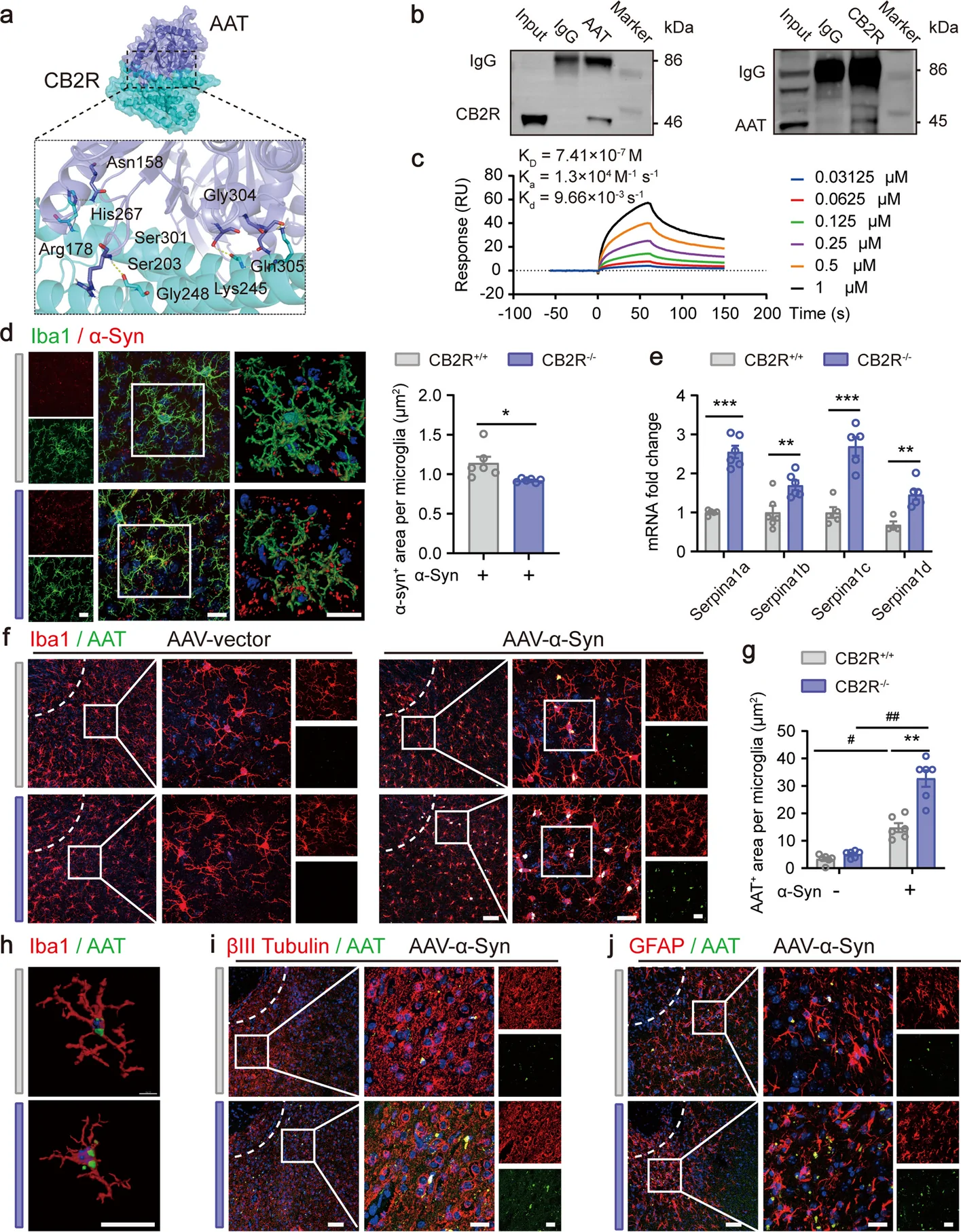
Complementary analyses support an AAT-CB2R association and reveal enhanced *Serpina1*/AAT responses under *Cnr2* deficiency. **a** Protein–protein docking model showing the predicted AAT-CB2R interaction interface, visualized using PyMOL. **b** Representative WB of the Co-IP of CB2R and AAT in mouse NAc extracts. **c** SPR kinetic analysis showing real-time, concentration-dependent binding of recombinant CB2R (0.03125–1 μM) to immobilized AAT. Sensorgrams represent reference-subtracted binding responses, with black overlay curves showing the global fit to a 1:1 Langmuir binding model. **d** IF staining of the NAc area using Iba1 (green) and α-syn (red) antibodies. Scale bar: 20 μm. Quantification of α-syn^+^ area per microglia (*n* = 6). **e** The mRNA expression of *Serpina1a*, *Serpina1b*, *Serpina1c*, and *Serpina1d* in AAV2/9-hSyn-*SNCA*(A53T)-WPRE-treated CB2R^+/+^ and CB2R^−/−^ mice (*n* = 4–6 per group; samples falling outside the A260/A280 RNA purity range of 1.8–2.0 were excluded from the analysis). *p < 0.05, **p < 0.01, ***p < 0.001, CB2R^+/+^ vs. CB2R^−/−^ mice injected with AAV-α-syn, by unpaired *t*-test. **f** IF staining with Iba1 (red) and AAT (green) in AAV-vector- or AAV-α-syn-treated CB2R^+/+^ and CB2R^−/−^ mice. scale bar: 50 μm, 20 μm. **g** Quantification of AAT^+^ area per microglia (*n* = 6). The data are presented as the mean ± SEM and assessed by two-way ANOVA followed by Tukey's multiple comparisons test. *p < 0.05, ^##^*p* < 0.01, ^###^*p* < 0.001, between mice treated with AAV-vector and AAV-α-syn; *p < 0.05, **p < 0.01, ***p < 0.001, compared between CB2R^+/+^ and CB2R^−/−^ mice. **h** The 3D reconstruction via Imaris displayed the colocalization of Iba1 (red) and AAT (green), Scale bar: 20 μm. **i**, **j** IF staining with GFAP (red)/βIII tubulin (red) and AAT (green). Scale bar: 50 μm, 20 μm. PDB ID: 1HP7 for α1-antitrypsin and 6KPF for cannabinoid receptor 2. SPR, surface plasmon resonance; Co-IP, co-immunoprecipitation; Iba1, Ionized calcium binding adaptor molecule-1; GFAP, glial fibrillary acidic protein

Having identified *Serpina1*/AAT as an early CB2R-associated response to α-syn, we next examined whether AAT and CB2R could associate across complementary structural, biochemical, and biophysical assays. Protein–protein docking predicted a candidate interaction interface involving multiple hydrogen-bonding contacts, including AAT Asn158 and CB2R His267 (Fig. 2a). Reciprocal co-immunoprecipitation (Co-IP) of NAc lysates from α-syn-treated mice recovered AAT and CB2R within the same immunoprecipitable complex (Fig. 2b). In surface plasmon resonance (SPR) experiments, recombinant CB2R injected over immobilized AAT generated concentration-dependent sensorgrams. Global fitting to a 1:1 Langmuir binding model yielded an association rate constant (*k*_*a*_) of 1.3 × 10^4^ M^−1^ s^−1^ and a dissociation rate constant (*k*_*d*_) of 9.66 × 10^–3^ s^−1^, resulting in an apparent equilibrium dissociation constant (*K*_*D*_) of 7.41 × 10–7 M (Fig. 2c). Together, these findings support an AAT–CB2R association under the respective assay conditions, without defining the physiological binding site, receptor-activation mechanism, or an allosteric mode of modulation.

To determine whether the enhanced *Serpina1*/AAT response was also evident beyond the acute 2-h transcriptomic screen, we examined an independent chronic model generated by AAV-mediated α-syn overexpression in the NAc of CB2R^+/+^ and constitutive CB2R^−/−^ mice. CB2R^−/−^ mice exhibited greater α-syn immunoreactivity associated with Iba1-positive microglia in the NAc shell than CB2R^+/+^ mice (Fig. 2d). RT-qPCR analysis further revealed higher levels of *Serpina1a*, *Serpina1b*, *Serpina1c*, and *Serpina1d* transcripts in the NAc of α-syn-overexpressing CB2R^−/−^ mice (Fig. 2e). Consistent with these transcriptional changes, AAT immunoreactivity was increased in CB2R^−/−^ mice and was detected predominantly in Iba1-positive cells under the conditions examined (Fig. 2f-h and Fig. S3a). In contrast, only limited overlap was observed between AAT immunoreactivity and the astrocytic marker GFAP or neuronal marker βIII-tubulin (Fig. 2i, j and Fig. S3b, c). These colocalization findings indicate a predominant association of AAT immunoreactivity with microglia but do not establish exclusive microglial synthesis or exclude the uptake of extracellular AAT.

The structural, biochemical, and biophysical findings therefore support an AAT–CB2R association under the respective experimental conditions. In the chronic AAV–α-syn model, constitutive *Cnr2* deficiency was accompanied by a greater microglia-associated α-syn burden and enhanced *Serpina1*/AAT responses in the NAc. The predominant association of AAT immunoreactivity with Iba1-positive cells, together with its increase under *Cnr2*-deficient conditions, is compatible with a compensatory response involving microglia during persistent α-syn-associated inflammation, although the present data do not establish this interpretation. Nor do these findings distinguish whether the increased *Serpina1*/AAT response reflects direct CB2R-related transcriptional regulation or an indirect consequence of the heightened inflammatory state associated with *Cnr2* deficiency. This evidence motivated subsequent experiments to determine whether CB2R-associated signaling contributes to the immunomodulatory effects of exogenous AAT.

### AAT attenuates microglial NLRP3/caspase-1-related inflammation through a CB2R-associated mechanism in an α-synucleinopathy model

**Fig. 3:**
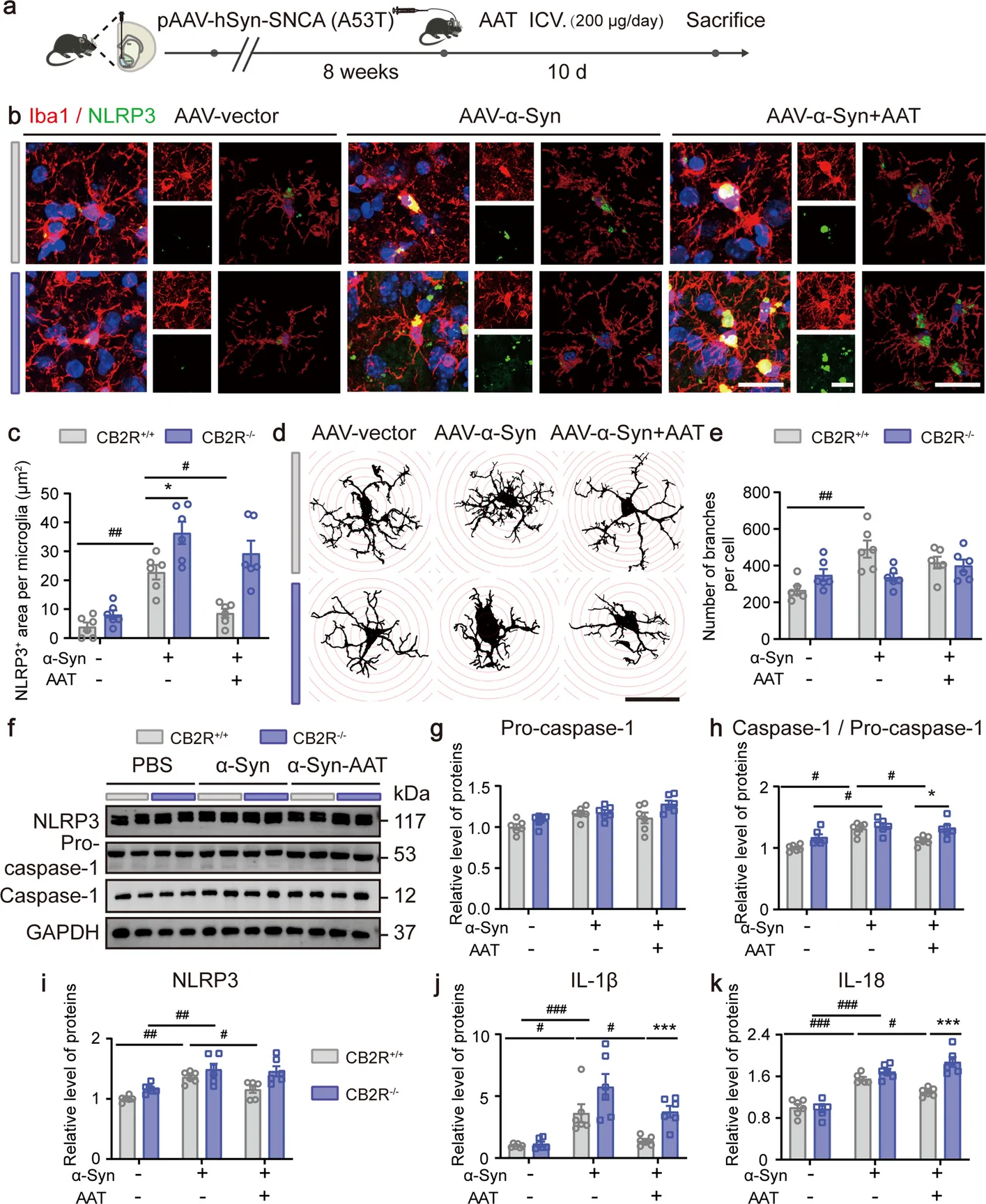
*Cnr2* deficiency diminishes the anti-inflammatory effects of AAT on microglial NLRP3/caspase-1-related signaling. **a** The schematic graph illustrates the experimental protocol. **b** IF staining for Iba1 (red) and NLRP3 (green) and the 3D reconstruction image via Imaris were performed in different group. Scale bar: 20 μm. **c** Data of the NLRP3^+^ fluorescence density per microglia (*n* = 6). **d** The sholl images of the microglial morphological changes for different groups, starting radius: 4 μm, radius step size: 3 μm, scale bar: 20 μm. **e** Sholl analysis and skeleton analysis of microglia showed the processes and branches between six groups. (*n* = 6). **f** Representative western blots and (**g**-**i**) densitometric analysis of NLRP3, pro-caspase-1, and caspase-1 expression in primary microglia. The different protein levels were normalized against GAPDH level (as a control) (*n* = 4). **j**, **k** ELISA assay revealed the changes in IL-1β and IL-18 level (*n* = 6). The data are presented as the mean ± SEM and assessed by two-way ANOVA followed by Tukey's multiple comparisons test. ^#^*p* < 0.05, ^##^*p* < 0.01, ^###^*p* < 0.001, between mice treated with AAV-vector, AAV-α-syn and AAV-α-syn + AAT; *p < 0.05, **p < 0.01, ***p < 0.001, compared between CB2R^+/+^ and CB2R.^−/−^ mice with different treatment. ICV, intra-cerebroventricular injection; IL-1β, interleukin-1β (*Il1b*); IL-18 (*Il18*), interleukin-18

Having obtained complementary evidence for an AAT–CB2R association, we next examined whether CB2R signaling contributed to the anti-inflammatory effects of exogenous AAT. In the chronic AAV–α-syn model, CB2R^+/+^ and constitutive CB2R^−/−^ mice received intracerebroventricular (ICV) AAT, after which microglial morphology and NLRP3-related inflammatory markers were evaluated in the NAc (Fig. 3a). Dual immunofluorescence staining for Iba1 and NLRP3, together with three-dimensional reconstruction, showed increased NLRP3 immunoreactivity in Iba1-positive cells following α-syn overexpression. AAT significantly reduced this signal in CB2R^+/+^ mice, whereas no comparable reduction was detected in CB2R^−/−^ mice (Fig. 3b, c). Sholl and skeleton analyses further revealed α-syn-associated alterations in microglial process number and branching complexity (Fig. 3d, e). In CB2R^+/+^ mice, AAT shifted these morphological measures toward the corresponding control values. In contrast, the effects of AAT were markedly attenuated or not detected in CB2R^−/−^ mice, in which microglia retained shorter, thicker processes and altered branching patterns after treatment (Fig. 3d, e and Fig. S4a, b). These observations indicate genotype-associated differences in microglial morphological remodeling, although static morphology alone does not define a specific functional or activation state.

We next assessed NLRP3/caspase-1-related inflammatory signaling in α-syn-stimulated primary microglia. AAT reduced α-syn-induced increases in NLRP3, pro-caspase-1, and caspase-1 protein abundance in CB2R^+/+^ microglia (Fig. 3f-i). These AAT-associated reductions were markedly attenuated or not detected in CB2R^−/−^ cells. Consistent with the protein findings, AAT also reduced α-syn-associated increases in IL-1β and IL-18 levels in CB2R^+/+^ microglia, whereas no comparable reductions were observed in CB2R^−/−^ counterparts (Fig. 3j, k). Overall, these findings support a substantial contribution of CB2R signaling to AAT-mediated effects on α-syn-associated microglial remodeling and NLRP3/caspase-1-related inflammatory responses, while also allowing for the involvement of parallel anti-inflammatory mechanisms.

### AAT attenuates α-syn-associated microglial Ca^2^⁺/calpain signaling and GSK-3β cleavage in a CB2R-associated manner

**Fig. 4:**
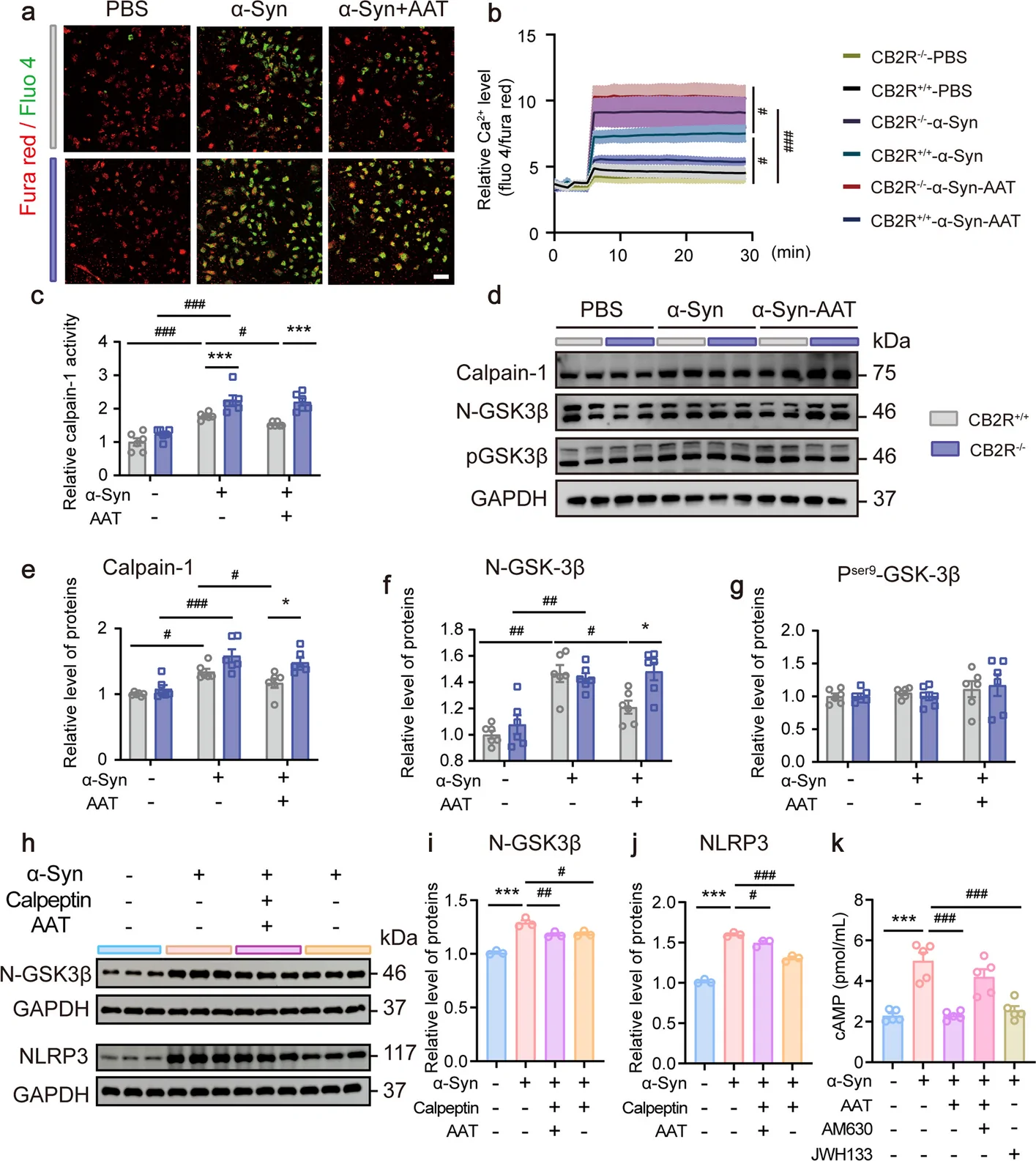
AAT modulates microglial Ca^2^⁺/calpain/GSK-3β-related signaling with attenuated responses under *Cnr2*-deficient conditions. **a** Representative time-lapse images of intracellular calcium (Ca^2^⁺) flux in primary microglia stained with a Ca^2^⁺-sensitive dye (Fura Red/Fluo-4) over 30 min. Scale bar: 50 μm. **b** Quantification of relative intracellular Ca^2^⁺ levels (Fluo-4/Fura Red fluorescence ratio) following ATP stimulation. (*n* = 4). **c** Relative calpain-1 activity in primary microglia isolated from CB2R^+/+^ and CB2R^−/−^ mice under the indicated treatment conditions (*n* = 6). **d**-**g** Representative WB (**d**) and densitometric quantification of calpain-1(**e**), N-terminal GSK-3β (N-GSK-3β) (**f**), and phosphorylated GSK-3β (Ser9) (**g**) protein levels, GAPDH was used as a loading control. (*n* = 4). The data are presented as the mean ± SEM and assessed by two-way ANOVA followed by Tukey's multiple comparisons test. ^#^*p* < 0.05, ^##^*p* < 0.01, ^###^*p* < 0.001, between primary microglia treated with PBS, α-syn, and α-syn + AAT; *p < 0.05, **p < 0.01, ***p < 0.001 indicate genotype comparisons between CB2R^+/+^ and CB2R^−/−^ microglia under the same treatment condition. **h**-**j** Representative WB (**h**) and densitometric quantification of N-GSK-3β (**i**) and NLRP3 (**j**) protein levels in WT primary microglia treated with α-syn, in the presence or absence of the calpain inhibitor calpeptin or AAT (*n* = 3). **k** Intracellular cAMP levels measured via ELISA under the indicated treatment conditions (*n* = 5). The data are presented as the mean ± SEM and assessed by one-way ANOVA followed by Tukey's multiple comparisons test. Gsk-3β, glycogen synthase kinase 3 beta; N-Gsk-3β, N-terminal glycogen synthase kinase 3 beta; P^ser9^-Gsk-3β, phospho-serine 9-glycogen synthase kinase 3 beta; cAMP, cyclic adenosine 3′,5′-monophosphate

Dysregulation of intracellular Ca^2^⁺ homeostasis has been implicated in microglial NLRP3 inflammasome-related signaling and the amplification of neuroinflammatory responses in neurodegenerative disorders [18, 19]. To determine whether AAT altered this response, we monitored ATP-evoked intracellular Ca^2^⁺ dynamics in α-syn-treated primary microglia using the Ca^2^⁺-sensitive fluorophores Fluo-4 and Fura Red. α-Syn treatment increased the ATP-evoked Fluo-4/Fura-Red fluorescence ratio, whereas AAT attenuated this increase in CB2R^+/+^ microglia. No comparable reduction was detected in CB2R^−/−^ cells (Fig. 4a, b). Similarly, AAT reduced α-syn-associated NLRP3 protein upregulation in CB2R^+/+^ microglia, whereas this effect was not detected in CB2R^−/−^ cells under the conditions examined (Fig. S5a, b).

Elevated cytosolic Ca^2^⁺ can activate calpain, a Ca^2^⁺-dependent cysteine protease that mediates N-terminal cleavage of glycogen synthase kinase-3β (GSK-3β), a proteolytic modification reported to enhance GSK-3β activity [20]. GSK-3β has also been implicated in the regulation of NLRP3 inflammasome-related signaling and neuroinflammatory responses in models of PD [21, 22]. We therefore examined whether calpain/GSK-3β signaling was associated with the CB2R-related effects of AAT in α-syn-stimulated microglia. α-Syn exposure increased both calpain-1 activity (Fig. 4c) and calpain-1 protein abundance (Fig. 4d, e). AAT attenuated both changes in CB2R^+/+^ microglia, whereas no significant reductions were detected in CB2R^−/−^ cells.

α-Syn exposure also increased the abundance of the N-terminally cleaved GSK-3β species, a proteolytic form previously associated with enhanced kinase activity (Fig. 4f). AAT reduced the abundance of this cleaved species in CB2R^+/+^ microglia but did not significantly alter it in CB2R^−/−^ cells. Because GSK-3β is also regulated by inhibitory phosphorylation at Ser9, we examined the abundance of Ser9-phosphorylated GSK-3β. No significant differences in the Ser9-phosphorylated species were detected among the experimental conditions (Fig. 4g). Thus, the measured AAT-associated effect was evident in GSK-3β N-terminal cleavage rather than in the Ser9 phospho-signal. Nevertheless, the potential involvement of other phosphorylation sites or regulatory mechanisms cannot be excluded.

To further examine the contribution of calpain-related signaling, α-syn-stimulated CB2R^+/+^ primary microglia were treated with the calpain inhibitor calpeptin. Calpeptin reduced both GSK-3β N-terminal cleavage and NLRP3 protein abundance, partially recapitulating the molecular effects of AAT (Fig. 4h-j). These findings are consistent with an upstream contribution of calpain to these molecular changes, although they do not establish GSK-3β as an obligatory intermediate linking calpain activity to NLRP3 regulation. We next measured intracellular cAMP as a downstream pharmacological readout relevant to CB2R signaling. AAT reduced the α-syn-associated elevation in intracellular cAMP, producing a response similar in direction to that observed with the CB2R agonist JWH133 (Fig. 4k). The AAT-associated reduction in cAMP was attenuated by the CB2R antagonist AM630, providing pharmacological evidence for the functional involvement of CB2R signaling under the experimental conditions examined. Together with the *Cnr2*-deficient experiments, these findings support a mechanistic framework in which CB2R-associated signaling contributes to AAT-mediated regulation of intracellular Ca^2^⁺ responses, calpain activity, GSK-3β N-terminal cleavage, and NLRP3-related inflammatory signaling.

**Fig. 5:**
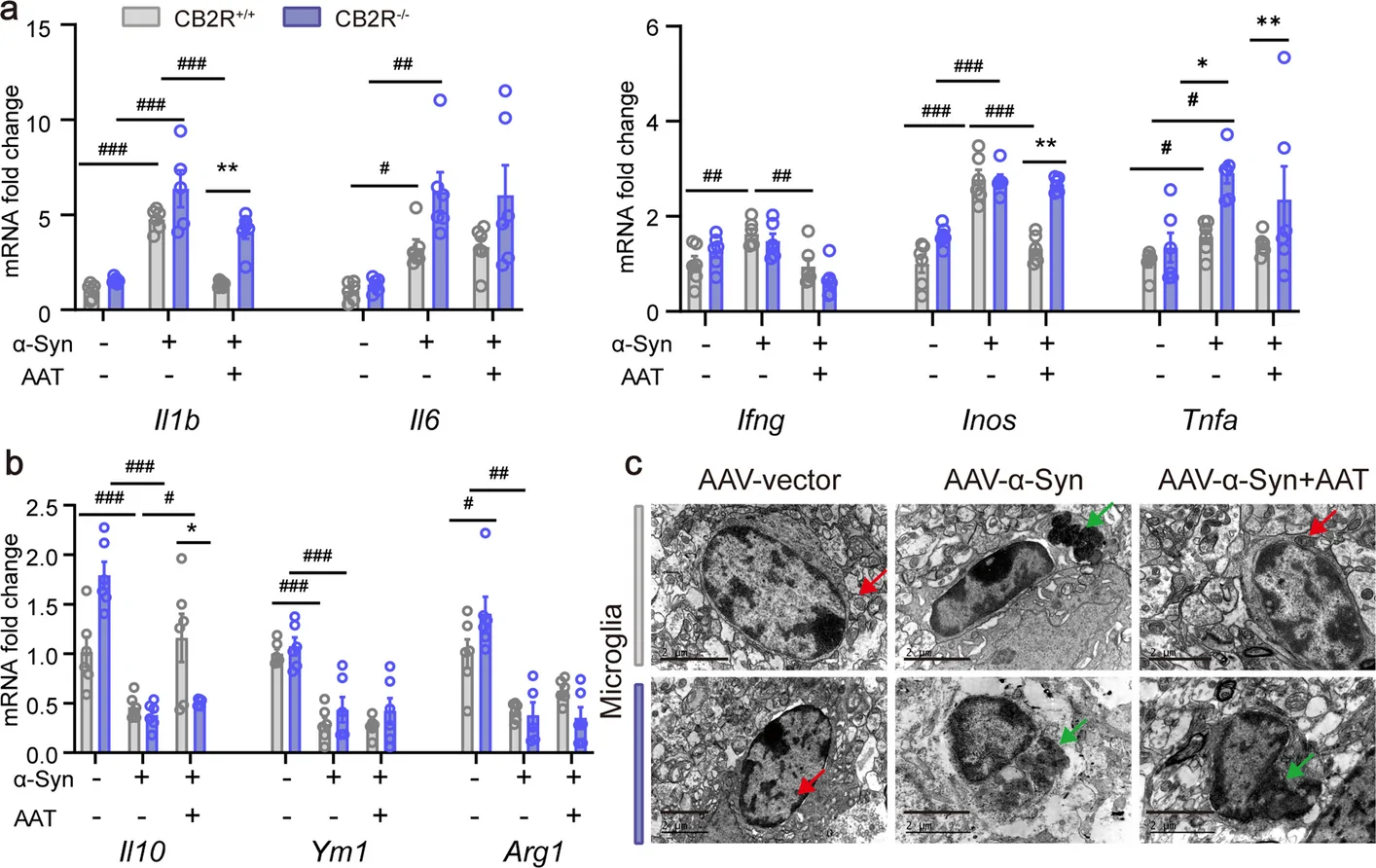
AAT modulates inflammatory gene expression and microglial organelle ultrastructure under α-syn-associated conditions. **a**, **b** RT-qPCR analysis of mRNA levels for pro-inflammatory (**a**; *Il1b*, *Il6*, *Ifng*, *Inos*, *Tnfa*) and anti-inflammatory (**b**; *Il10*, *Ym1*, *Arg1*) markers in primary microglia isolated from CB2R^+/+^ and CB2R^−/−^ mice under the indicated treatment conditions (*n* = 6). **c** Representative TEM images of microglia in the NAc across different AAV treatment groups. Red arrows indicate mitochondria, and green arrows indicate lysosomes. Scale bar, 2 μm. The data are presented as the mean ± SEM and assessed by two-way ANOVA followed by Tukey's multiple comparisons test. ^#^*p* < 0.05, ^##^*p* < 0.01, ^###^*p* < 0.001, between primary microglia treated with PBS, α-syn, and α-syn + AAT; *p < 0.05, **p < 0.01, ***p < 0.001 indicate genotype comparisons between CB2R^+/+^ and CB2R.^−/−^ microglia under the same treatment condition. IL-6 (*Il6*), interleukin-6; IL-10 (*Il10*), interleukin-10; IFN-γ (*Ifng*), interferon-γ; iNOS (*Inos*), inducible nitric oxide synthase; TNF-α (*Tnfa*), tumor necrosis factor-alpha; Ym1/Chi313 (*Ym1*), chitinase-3-like protein 3; Arg-1, arginase-1

Broader inflammatory transcriptional responses were subsequently profiled in α-syn-stimulated microglia. In CB2R^+/+^ cells, AAT attenuated the α-syn-induced increases in *Il1b*, *Ifng*, *Inos*, and *Tnfa* mRNA abundance and shifted *Il10* mRNA abundance toward control levels. These transcriptional effects were markedly attenuated or not detected in CB2R^−/−^ microglia (Fig. 5a, b), providing further evidence that CB2R-associated signaling contributes to the immunomodulatory actions of AAT. Ultrastructural examination by transmission electron microscopy further suggested comparatively better preservation of mitochondrial morphology and less pronounced alterations in lysosome-like vesicle profiles in NAc microglia from AAT-treated CB2R^+/+^ mice. Similar qualitative improvements were not evident in CB2R^−/−^ mice, in which mitochondrial abnormalities and altered lysosome-like vesicle profiles remained apparent following AAT administration (Fig. 5c). As these observations were based on static, representative electron micrographs without quantitative or functional assessment, they provide descriptive evidence of genotype-associated differences in organelle ultrastructure but do not establish enhanced lysosomal biogenesis, restoration of lysosomal function, or irreversible organelle injury.

Taken together, the genetic, pharmacological, molecular, and qualitative ultrastructural findings provide convergent evidence that CB2R-associated signaling contributes substantially to the effects of AAT on α-syn-associated intracellular Ca^2^⁺ responses, calpain activity, GSK-3β N-terminal cleavage, NLRP3-related inflammatory signaling, and microglial organelle morphology. These results support a contributory CB2R-associated mechanism while allowing for the involvement of parallel CB2R-independent pathways.

### CB2R signaling contributes to AAT-mediated improvement of selected excitatory synaptic abnormalities in NAc D2-MSNs during α-syn pathology

**Fig. 6:**
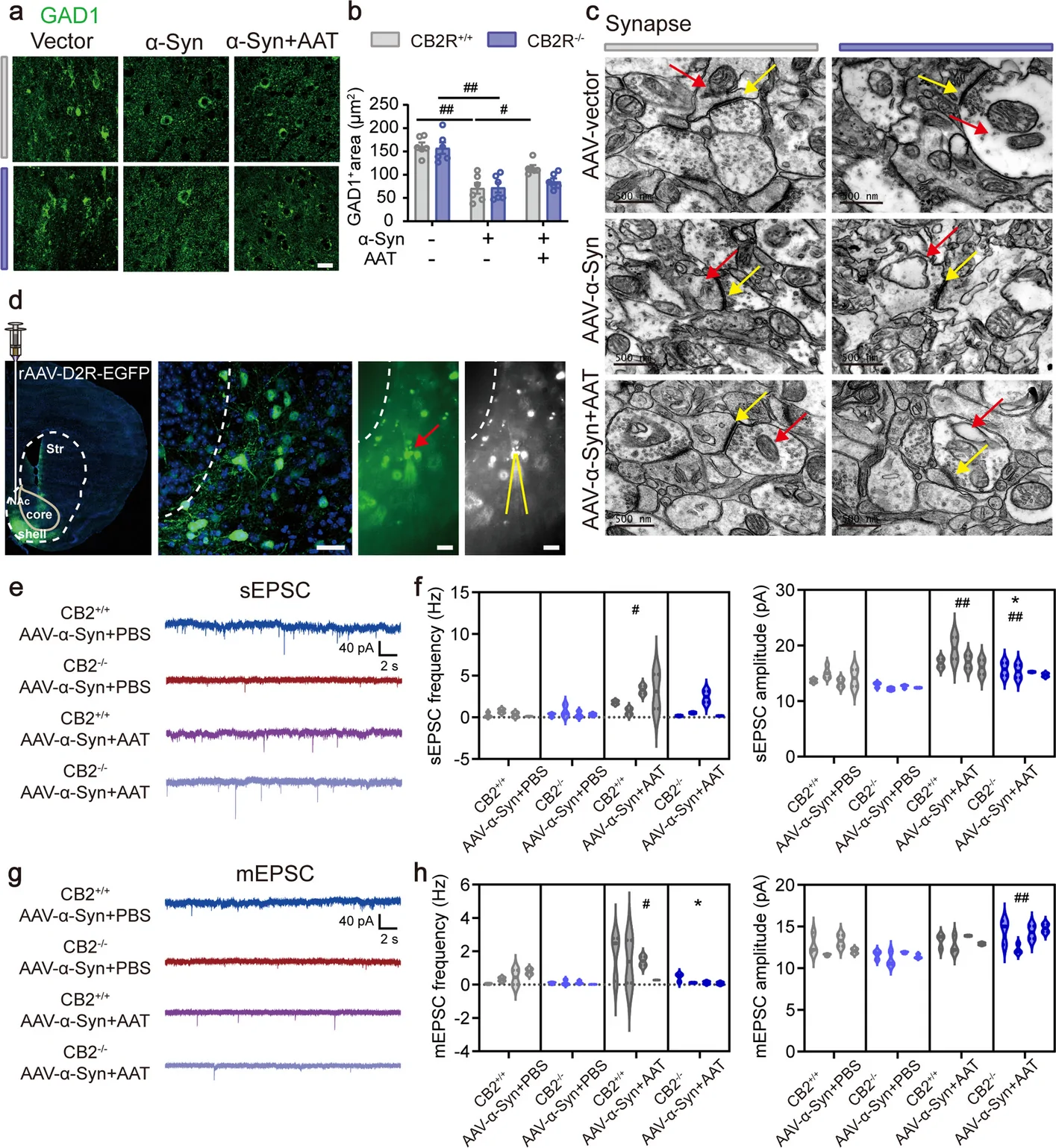
AAT mitigates neuronal damage and restores synaptic transmission in NAc D2-MSNs. **a** Representative immunofluorescence images of GAD1 (green) in the NAc. Scale bar: 20 µm. **b** Quantification of the GAD1⁺ area (*n* = 6). The data are assessed by two-way ANOVA followed by Tukey's multiple comparisons test. **c** Representative TEM images of NAc synapses. Red and yellow arrows indicate mitochondria and the synaptic cleft, respectively (*n* = 3), scale bar: 500 nm. **d** Merged fluorescence (D2R, green; DAPI, blue) and bright-field images delineating the stereotaxic injection area of AAV-D2R-EGFP and the patch-clamp recording site within the NAc shell. **e**, **g** Representative traces of sEPSCs (**e**) and mEPSCs (**g**) recorded from GFP-positive D2-MSNs across the four experimental groups. **f**, **h** Quantitative analyses of the frequency (left) and amplitude (right) of sEPSCs (**f**) and mEPSCs (**h**). Data are presented as mean ± SEM (*n* = 8–11 cells nested within *N* = 4 mice per group). Statistical significance was assessed by nested ANOVA followed by Tukey’s multiple comparisons test. ^#^*p* < 0.05, ^##^*p* < 0.01, and ^###^*p* < 0.001 for comparisons between PBS, AAV-α-Syn + AAT and AAV-α-Syn + PBS within the same genotype; *p < 0.05, **p < 0.01, and ***p < 0.001 for comparisons between genotypes (CB2R^−/−^ vs. CB2R^+/+^) under identical treatments. D2-MSNs, dopamine receptor type 2-expressing medium spiny neurons; TEM, transmission electron microscopy; GAD1, glutamate decarboxylase 1; sEPSC, spontaneous excitatory postsynaptic current; mEPSC, miniature excitatory postsynaptic currents

Given that microglial inflammatory signaling can influence neighboring neuronal circuits, we next examined neuronal and synaptic alterations within the NAc. GABAergic medium spiny neurons constitute the principal neuronal population in this region and integrate dopaminergic and glutamatergic inputs that shape NAc circuit output. In α-syn-overexpressing mice, immunoreactivity for glutamate decarboxylase 1 (GAD67), the 67-kDa protein encoded by GAD1, was reduced in the NAc, consistent with an altered GABAergic neuronal phenotype, although this measure does not directly establish impaired MSN output function (Fig. 6a, b). Representative transmission electron micrographs further revealed features suggestive of altered synaptic morphology and mitochondrial ultrastructure, including less well-defined synaptic clefts and disrupted mitochondrial membrane and cristae profiles. These abnormalities appeared more pronounced in CB2R^−/−^ mice (Fig. 6c). These static observations provide qualitative evidence of ultrastructural differences but do not independently establish synaptic failure or mitochondrial dysfunction.

NAc D2-MSNs participate in the processing of aversive and motivationally relevant signals and depend on excitatory inputs from cortical and limbic circuits [23]. We therefore examined whether AAT treatment was associated with changes in excitatory synaptic input onto D2-MSNs in the NAc shell. Whole-cell patch-clamp recordings were performed in EGFP-positive D2-MSNs to measure spontaneous and miniature excitatory postsynaptic currents (sEPSCs and mEPSCs; Fig. 6d-h). Electrophysiological data were analyzed using nested ANOVA, with individual cells nested within their respective animals to account for the hierarchical structure of the dataset. The analysis included 8–11 cells from four mice per group.

In α-syn-overexpressing CB2R^+/+^ mice, AAT significantly increased both sEPSC and mEPSC frequencies relative to vehicle-treated α-syn controls. Corresponding frequency increases were not detected in CB2R^−/−^ mice (Fig. 6f, h), supporting a substantial contribution of CB2R signaling to the frequency-related effects of AAT on excitatory synaptic input. In contrast, AAT increased sEPSC amplitude in both genotypes and increased mEPSC amplitude in CB2R^−/−^ mice. These genotype-divergent amplitude responses indicate that selected electrophysiological effects of AAT persist in the absence of CB2R.

Collectively, these findings show that AAT ameliorates selected α-syn-associated abnormalities in excitatory synaptic transmission onto NAc D2-MSNs. The genotype dependence of the sEPSC and mEPSC frequency responses supports the involvement of CB2R-associated signaling, whereas the retained amplitude-related responses in CB2R^−/−^ mice suggest that complementary CB2R-independent mechanisms may also contribute.

### AAT attenuates affective and domain-specific cognitive impairments in an α-synucleinopathy model with a substantial contribution from CB2R signaling

**Fig. 7:**
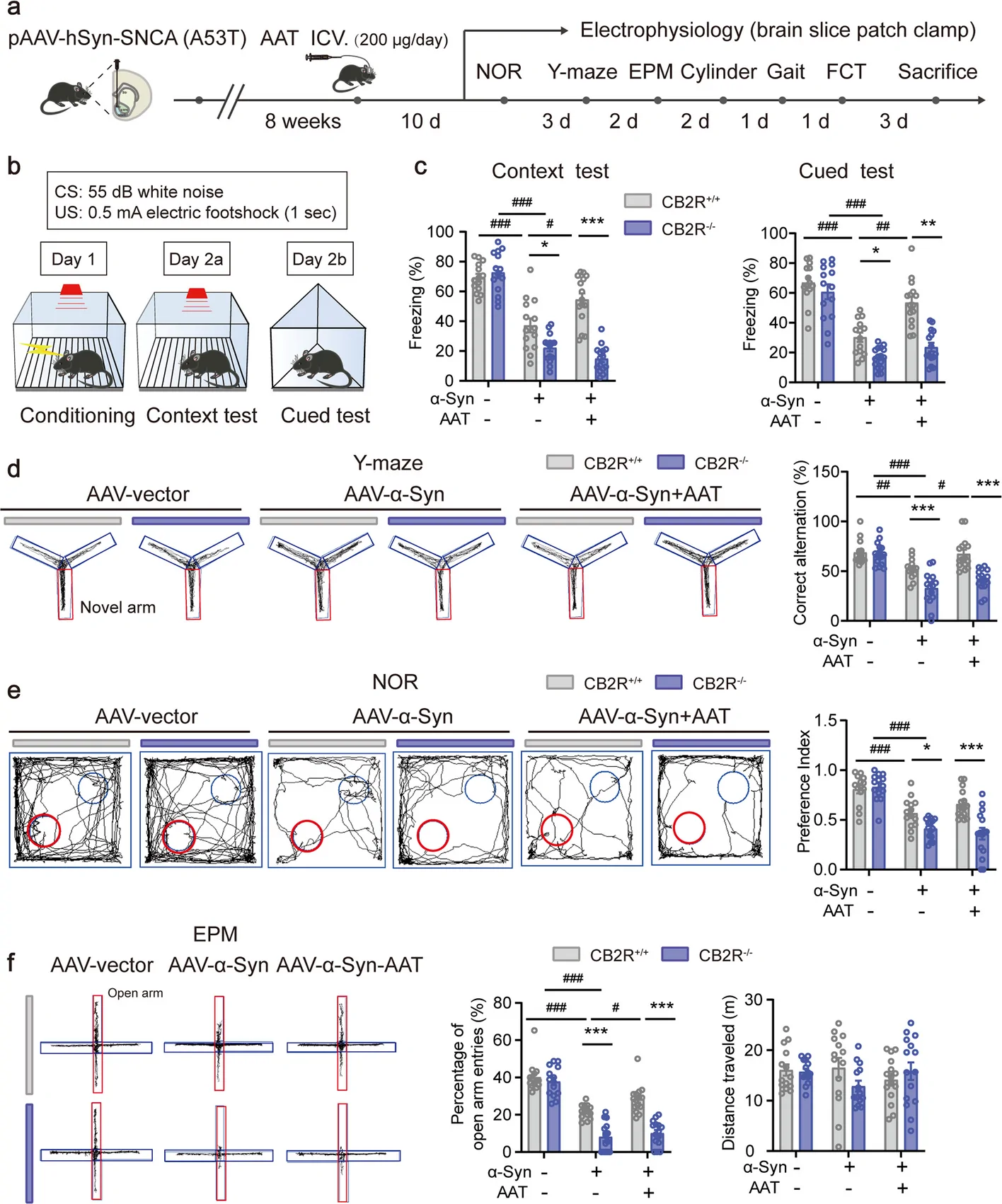
AAT ameliorates affective and domain-specific cognitive deficits in α-synucleinopathy mice. **a** The schematic graph illustrates the experimental protocol. **b** Schema of the contextual and cued test fear condition, day 1: conditioning; day 2a: context test; day 2b: cued test. **c** The percentage of freezing time in mice subjected to contextual and cued fear conditioning tests. **d** The alternation percentage of novel arm (red) observed in mice during the Y-maze assay. **e** The observed preference index in mice during the NOR test. **f** Representative tracking traces of EPM and quantification of the frequency of entries into the open arm (red) and total distance traveled. The data are presented as the mean ± SEM and assessed by two-way ANOVA followed by Tukey's multiple comparisons test (*n* = 14–15). ^#^*p* < 0.05, ^##^*p* < 0.01, ^###^*p* < 0.001, between mice treated with AAV-vector, AAV-α-syn, and AAV-α-syn + AAT; *p < 0.05, **p < 0.01, ***p < 0.001, compared between CB2R^+/+^ and CB2R^−/−^ mice with different treatment. FCT, fear conditioning test; EPM, elevated plus maze; NOR, novel object recognition

To evaluate the functional consequences of AAT treatment and determine whether the molecular and synaptic changes described above were accompanied by behavioral improvement, mice with NAc-targeted A53T α-syn overexpression were subjected to a comprehensive behavioral battery assessing associative fear memory, spatial working-memory-related performance, recognition memory, anxiety-related behavior, and motor function (Fig. 7a). α-Syn-overexpressing mice exhibited significant impairments in associative fear memory, as evidenced by reduced freezing during both contextual and cued retrieval tests compared with vector-injected controls (Fig. 7b, c). These deficits were more pronounced in CB2R^−/−^ mice than in CB2R^+/+^ littermates, indicating greater behavioral vulnerability in the absence of CB2R signaling.

In CB2R^+/+^ mice, AAT significantly increased freezing during both contextual and cued retrieval, shifting performance toward the corresponding control levels. AAT also improved Y-maze alternation performance, consistent with enhanced spatial working-memory (Fig. 7d). In contrast, AAT did not significantly alter novel object recognition performance (Fig. 7e) or the gait and cylinder-test measures examined (Fig. S6a, b). While, in the elevated plus maze, AAT increased open-arm entries, reflecting reduced anxiety-like behavior (Fig. 7f). These behavioral benefits of AAT were markedly attenuated or absent in CB2R^−/−^ mice, indicating domain-selective rather than generalized effects.

The behavioral improvements observed in CB2R^+/+^ mice occurred in parallel with the attenuation of microglial NLRP3/caspase-1-related inflammatory signaling and genotype-sensitive changes in excitatory synaptic input onto NAc D2-MSNs described above. Although the present experimental design does not establish direct mediation among these molecular, synaptic, and behavioral outcomes, their convergence supports a functional contribution of CB2R-associated signaling to several AAT-mediated effects. Thus, AAT attenuated selected affective and cognitive impairments associated with NAc α-syn pathology, with CB2R signaling contributing substantially to the observed behavioral benefits.

## Discussion

**Fig. 8:**
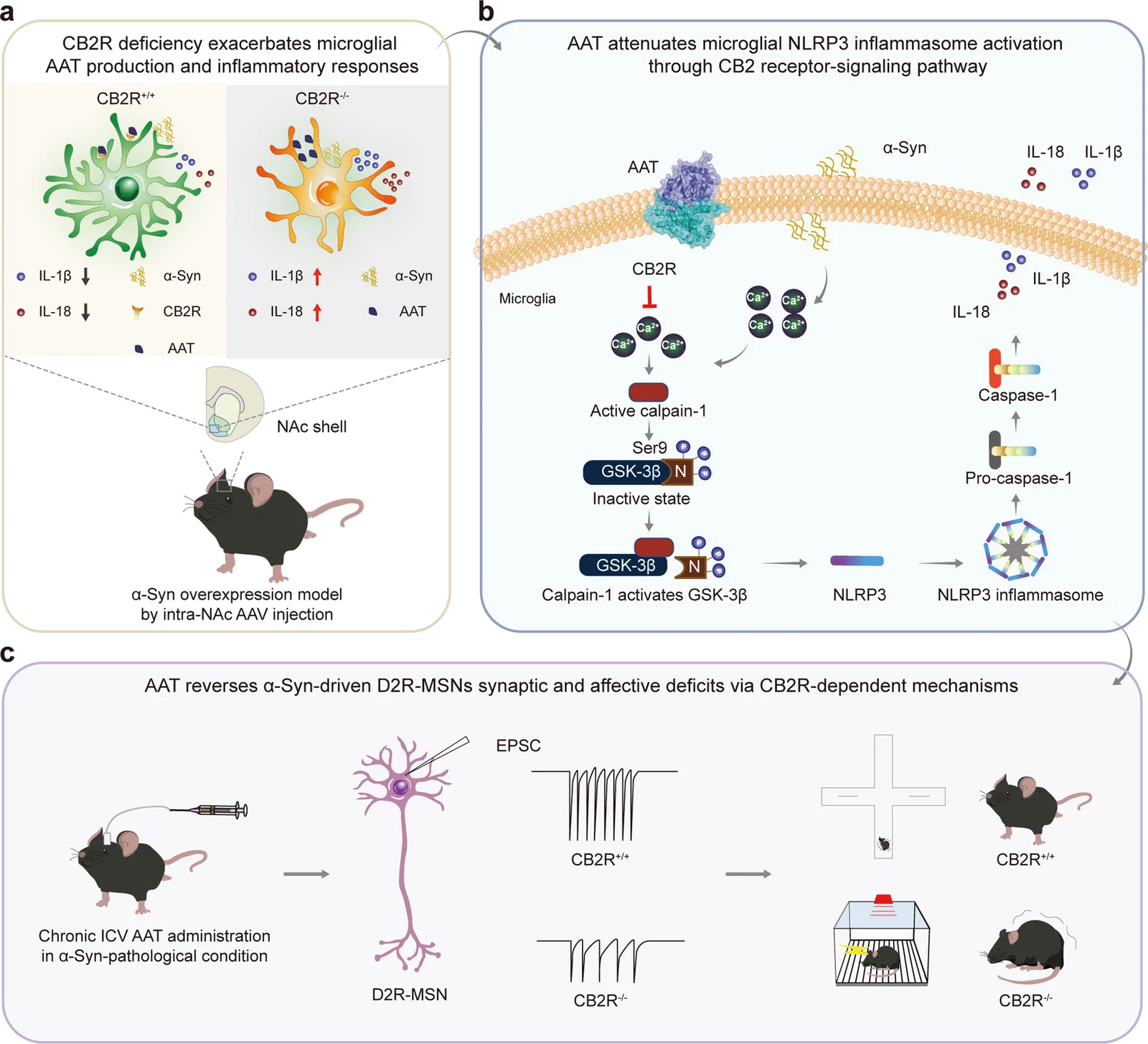
Proposed model of AAT-mediated modulation of CB2R-linked neuroimmune signaling in α-syn pathology. Panel **a** demonstrates that α-syn overexpression in the NAc shell elevates microglial AAT production and induces inflammatory responses. While CB2R deficiency further enhances microglial AAT expression yet exacerbates the persistent release of pro-inflammatory cytokines (IL-1β, IL-18). Panel **b** reveals AAT-CB2R-linked signaling contributes to the attenuation of ATP-evoked intracellular Ca^2^⁺ responses, calpain activity, GSK-3β N-terminal cleavage, and NLRP3/caspase-1-related inflammatory signaling, while complementary CB2R-independent actions may also contribute to its broader immunomodulatory effects. Panel **c** shows that at the circuit and behavioral levels, AAT administration ameliorates selected excitatory synaptic abnormalities in NAc dopamine D2-MSNs and improves domain-selective affective and cognitive outcomes associated with α-syn pathology

This study identifies AAT as a previously unrecognized modulator of CB2R-associated neuroimmune signaling in an NAc-centered α-synucleinopathy model (Fig. 8). Exogenous AAT attenuated microglial NLRP3/caspase-1-related inflammation and Ca^2^⁺/calpain-associated GSK-3β cleavage while improving selected D2-MSN synaptic abnormalities and domain-selective affective and cognitive phenotypes. Molecular association studies, *Cnr2*-deficient experiments, and AM630-sensitive cAMP modulation support a functional contribution of CB2R signaling, whereas the persistence of selected electrophysiological effects indicates complementary CB2R-independent actions.

AAT is an acute-phase glycoprotein with canonical antiprotease and broader immunoregulatory functions [14]. Acute-phase dysregulation has been reported in parkinsonian disorders [16], and AAT can restrain microglia-mediated inflammation under neurodegenerative stress [17]. Extending our previous finding that *Cnr2* deficiency aggravates α-syn-associated microglial and synaptic pathology in the NAc [13], α-syn exposure induced a prominent *Serpina1*/AAT response that was further amplified under *Cnr2*-deficient conditions. Its predominant localization to Iba1-positive cells identifies microglia as the principal cellular compartment associated with this response, although local synthesis cannot be distinguished from uptake or retention of extracellular AAT. The amplified response may represent an insufficient compensatory reaction to persistent inflammation. This interpretation also reconciles the reduction of endogenous AAT immunoreactivity by JWH133 with the anti-inflammatory effects of administered AAT: CB2R activation may reduce the inflammatory drive for endogenous AAT induction, whereas exogenous AAT may reinforce immunoregulation through both CB2R-associated and canonical antiprotease mechanisms [14, 17].

The acute and chronic models provide complementary but distinct evidence. The 2-h transcriptomic screen nominated the *Serpina1* family as a component of the early NAc response to α-syn, whereas the chronic AAV–α-syn model tested the mechanistic and therapeutic relevance of AAT after inflammatory, synaptic, and behavioral abnormalities had emerged. Persistent α-syn can sustain innate immune activation and impede inflammatory resolution [24]. Nevertheless, the present design does not establish that the acute *Serpina1* response progresses directly into the 8-week phenotype; this possibility requires longitudinal testing within a unified model.

Docking, Co-IP, and SPR support an AAT–CB2R association at complementary levels. Docking predicted a candidate interface, Co-IP placed both proteins within the same immunoprecipitable complex, and SPR demonstrated concentration-dependent binding of recombinant CB2R to immobilized AAT under cell-free conditions. These data do not define the physiological binding site or mode of receptor modulation. Peptide and noncanonical cannabinoid-receptor regulation provide a conceptual precedent [25], but an extracellular or allosteric AAT–CB2R interaction remains to be tested directly. Functional evidence was provided by the attenuation of multiple AAT responses under *Cnr2* deficiency and by the AM630-sensitive cAMP response, consistent with the established immunomodulatory actions of CB2R in the CNS [10, 11, 26]. Because cAMP was measured as a downstream endpoint, however, the data do not establish conventional CB2R agonism or canonical Gi/o coupling.

Within this framework, AAT attenuated ATP-evoked Ca^2^⁺ signaling, calpain-1 activity and abundance, and GSK-3β N-terminal cleavage, whereas Ser9 phosphorylation was unchanged. Calpain-mediated truncation can enhance GSK-3β activity [27], and calpeptin reduced both GSK-3β cleavage and NLRP3 abundance, supporting an upstream contribution of calpain. The requirement for GSK-3β cleavage in NLRP3 regulation was not directly tested, and NLRP3 integrates multiple ionic, metabolic, organelle, and transcriptional inputs [19, 21, 22]. These findings support a contributory Ca^2^⁺/calpain/GSK-3β mechanism within a broader inflammatory response rather than a single obligatory cascade. AAT’s established antiprotease and immunoregulatory properties [14, 17] may also explain why selected responses persisted under *Cnr2*-deficient conditions.

This mechanistic heterogeneity extended to the NAc synaptic and behavioral phenotypes. CB2R can modulate mesolimbic excitability, whereas D1- and D2-MSNs integrate cortical and limbic glutamatergic inputs relevant to motivational and affective processing [23, 28–31]. AAT was associated with relative preservation of NAc synaptic and mitochondrial ultrastructure and increased sEPSC and mEPSC frequencies in D2-MSNs of *Cnr2*^+/+^ mice. Selected amplitude-related effects persisted under *Cnr2* deficiency, indicating differential receptor involvement across electrophysiological endpoints. At the systems level, AAT improved contextual and cued fear-memory retrieval, spontaneous alternation, and anxiety-like behavior without altering novel object recognition or the motor measures examined. These convergent findings support a neuroimmune contribution to domain-selective behavioral improvement but do not establish direct mediation by microglial NLRP3 signaling or an NAc-restricted circuit.

Several limitations define this interpretation. Constitutive global *Cnr2* deletion cannot resolve cell-specific receptor functions and may introduce developmental adaptation. ICV administration does not confine AAT exposure to the NAc, and its CNS biodistribution and pharmacokinetics were not determined. The acute and chronic models were not linked longitudinally; receptor-binding site and transducer coupling remain undefined; ultrastructural observations were primarily qualitative; and only male mice were studied. Future work should combine cell-selective genetics, direct receptor pharmacology, longitudinal models, and quantitative structural analyses. Although clinical AAT augmentation establishes the feasibility of repeated administration [32], CNS translation will require less invasive delivery and rigorous assessment of brain exposure, stability, safety, and sustained efficacy. Intranasal or carrier-assisted strategies represent testable options rather than established solutions [33].

In summary, AAT emerges as a multifunctional modulator of CB2R-associated neuroimmune signaling that attenuates inflammatory, synaptic, and non-motor consequences of α-syn pathology. These findings provide a mechanistic rationale for further cell-selective and translational evaluation of AAT-based interventions.

## Materials and methods

### Animals

*Cnr2*-knockout mice (B6.129P2-*Cnr2*^tm1Dgen^/J; RRID: IMSR_JAX:005786) were originally obtained from The Jackson Laboratory (Bar Harbor, ME, USA). Experimental male CB2R^+/+^ and CB2R^−/−^ littermates were generated and maintained as previously described [13]. Genotypes were determined by PCR analysis of tail-biopsy DNA using the primers listed in Table S1, with representative genotyping results shown in Fig. S1b. Mice were housed under a 12-h light/12-h dark cycle at 25 ± 1 °C and approximately 55% relative humidity, with ad libitum access to food and water. Within each genotype, mice were randomly assigned to the indicated experimental groups, with group sizes specified in the corresponding figure legends. Behavioral assessments were performed by investigators blinded to treatment allocation. At the experimental endpoint, mice were euthanized by cervical dislocation in accordance with the approved animal protocol. All animal procedures were approved by the Animal Care and Use Committee of Fujian Medical University (FJMU IACUC 2021–0354) and conducted in accordance with the approved institutional guidelines.

### RNA sequencing (RNA-seq)

Total RNA was extracted from NAc tissues using TRIzol® Reagent. RNA quality and quantity were assessed before library preparation, and qualified samples were processed by Shanghai Majorbio Bio-pharm Biotechnology Co., Ltd. (Shanghai, China). RNA-seq libraries were prepared from 1 μg of total RNA using the Illumina® Stranded mRNA Prep kit and sequenced on an Illumina NovaSeq 6000 platform. Gene abundance was quantified using RSEM, and differential expression between predefined experimental groups was analyzed exclusively using the R package DESeq2. Differentially expressed genes (DEGs) were defined using the predefined thresholds of ∣log2FC∣ ≥ 1 and false discovery rate (FDR) ≤ 0.05. Gene Ontology (GO) and Kyoto Encyclopedia of Genes and Genomes (KEGG) pathway enrichment analyses were performed using GOATOOLS and KOBAS, respectively, with all detected genes used as the background set. Enriched terms and pathways with Bonferroni-corrected P ≤ 0.05 were considered significant.

### Stereotaxic surgery and adeno-associated viruses (AAV) injection

Eight-week-old male CB2R^+/+^ and CB2R^−/−^ mice were anesthetized with 1.5% isoflurane in 30% O_2_/68.5% N_2_O (1–2 L/min) and subjected to bilateral stereotaxic injections. Corneas were protected with ophthalmic ointment throughout the procedure. For the chronic α-syn model, AAV2/9-hSyn-SNCA(A53T)-WPRE (Heyuan; 2× 10^13^ gp/mL, 1 μL/site), encoding human A53T α-syn, was bilaterally injected into the NAc. For the acute α-syn exposure paradigm, recombinant fibrillar A53T α-syn (rPeptide, S-1002–2) was injected bilaterally at 1 μL/site and 0.1 μL/min. Stereotaxic coordinates relative to bregma were AP + 1.2 mm, ML ± 0.75 mm, and DV − 4.5 mm. For electrophysiological recordings, rAAV2/9-D2R-eGFP (Braincase) was subsequently injected to label D2-MSNs. Postoperative care included systemic meloxicam (1 mg/kg) for 3 consecutive days together with topical analgesic and antibiotic treatment.

### AAT administration

Eight weeks post-virus injection, mice underwent ICV guide cannula (62,003, RWD Life Science, Shenzhen) implantation under anesthesia. A 0.5 mm burr hole was drilled, and the cannula stereotaxically positioned at AP 0.8 mm, ML ± 1 mm, DV −1.5 mm (dura reference; insertion tip extended 0.5 mm beyond guide). The assembly was secured with dental resin (iTENA, France). After 3-day recovery, mice received daily ICV infusions of either AAT (Sigma A6150; 200 µg/day in 0.5 µL/min) or PBS during 22-day behavioral testing.

### Electrophysiological recordings

Following deep anesthesia,mice underwent transcardial perfusion with oxygenated ice-cold NMDG solution (components in supplemental files). Coronal slices (250 μm) containing the NAc were sectioned in ice-cold NMDG solution using a Leica VT1200s vibratome, then recovered at 34 °C for 15–30 min. Slices were perfused with oxygenated ACSF (25°C) at ~ 3 mL/min and visualized under infrared optics (60 × water-immersion lens). AAV-D2R-EGFP-infected neurons in NAc were identified by location and fluorescence. Whole-cell recordings of sEPSCs/mEPSCs used pipettes filled with internal solution. Additionally, the gamma-aminobutyric acid (GABA) type A receptors were blocked with bicuculline (10 μM), and TTX (1 μM) was added for mEPSCs recordings. Events were detected automatically (amplitude threshold: 10 pA) at − 70 mV holding potential. Signals were acquired with a MultiClamp 700B amplifier, filtered at 1 kHz, and sampled at 5 kHz (Digidata 1440 A, Clampex 10.2). Recordings were valid if series resistance remained within 15% of initial values (25–35 MΩ). mEPSCs were analyzed (MiniAnalysis 6.07) over 5-min epochs. Data are mean ± SEM.

### Behavioral tests

All behavioral assessments of motor and non-motor functions were conducted during the light phase between 10:00 and 16:00 under controlled dim red light conditions (15 lx). These behavioral experiments were conducted by examiners blinded to the groups, ensuring unbiased and objective evaluations. The details were described in the supplemental Information.

### Reverse transcription quantitative PCR (RT-qPCR)

Brain tissues from the nucleus accumbens and primary microglial cultures were homogenized and extracted using Trizol. Total RNA was extracted using a standardized protocol, followed by cDNA synthesis and SYBR Green-based quantitative PCR (qPCR) as previously described [13]. RNA concentration and purity were assessed spectrophotometrically. Samples with inadequate RNA purity (A260/A280 outside 1.8–2.0), failed amplification, or nonspecific amplification profiles were excluded from the final analysis. One microliter of cDNA was used with primer pairs listed in Table S2. qPCR was conducted on a Step One Plus Real-Time PCR System with the following protocol: 55 °C for 2 min, 95 °C for 10 min (1 cycle), 95 °C for 15 s, 60 °C for 1 min (40 cycles), and 95 °C for 15 s. Relative mRNA expression levels were determined using the 2-ΔΔCt method and normalized to GAPDH.

### Western blot and co-immunoprecipitation (Co-IP)

Brain and cell samples were lysed in RIPA buffer (Beyotime, P0013, China) with protease inhibitors (MCE, HY-K0010, USA), then analyzed by quantitative immunoblotting with primary antibodies (Table S3). Blots were visualized by ECL and quantified using Fiji software. For Co-IP, lysates were incubated overnight at 4 °C with anti-CB2R antibody, anti-AAT antibody, or control IgG (Table S3) bound to protein A/G magnetic beads (Thermo, #88,802, USA). After three lysis buffer washes, immunoprecipitated proteins were eluted by boiling (95 °C, 10 min) in SDS buffer, resolved by SDS-PAGE, and immunoblotted. Experiments included three biological replicates with technical triplicates, with representative blots shown.

### Surface plasmon pesonance (SPR) analysis

SPR assays were performed at 25 ℃ using a Biacore 1 K system (Cytiva, Marlborough, MA, USA) with 1 × PBS-P + (pH 7.4) as the running buffer. Purified AAT (ligand, ~ 52 kDa) was immobilized on a CM5 sensor chip (BR-1005–30, Cytiva) via standard amine coupling using an Amine Coupling Kit (BR-1000–50, Cytiva). The active flow channel was activated with a 1:1 mixture of 1-ethyl-3-(3-dimethylaminopropyl) carbodiimide and N-hydroxysuccinimide at a flow rate of 10 μL/min. AAT was diluted to 50 μg/mL in 10 mM sodium acetate buffer (pH 5.0) and immobilized on the activated surface at 10 μL/min, after which residual reactive groups were blocked with ethanolamine. A reference channel was activated and blocked in parallel without protein immobilization.

Recombinant human CB2R (analyte, ~ 42.5 kDa; Huamei Biotechnology, CSB-CF005679HU) was serially diluted (0.03125, 0.0625, 0.125, 0.25, 0.5, and 1 μM) in 95-well plates using interaction buffer (1 × PBS-P +, pH 7.4, containing 5% v/v DMSO). In a multi-cycle format, analyte solutions were sequentially injected over both active and reference channels from low to high concentration at a flow rate of 30 μL/min, with an association phase of 150 s per cycle. After each concentration injection, the sensor surface was regenerated with 10 mM glycine–HCl (pH 2.0) for 5 min. Responses from the reference channel were subtracted from those of the active channel and analyzed using Biacore Insight Evaluation software (Cytiva). The concentration-dependent sensorgrams were globally fitted to a 1:1 Langmuir binding model to derive the association-rate constant (*k*_*a*_), dissociation-rate constant (*k*_*d*_), and equilibrium dissociation constant (*K*_*D*_).

### PyMOL analysis

The crystal structures of AAT (1HP7) and CB2R (6KPF) were obtained from the PDB. Proteins were prepared using AutoDockTools-1.5.7 by removing water molecules and adding polar hydrogens. Protein–protein docking was performed with the GRAMM web server, and the resulting complex was further optimized. Interaction analysis and visualization were conducted in PyMOL, identifying key residues such as Asn158 (AAT) and His267 (CB2R) involved in hydrogen bonding. The top-ranked AAT–CB2R docking pose yielded a docking score of − 557.

### Transmission electron microscopy (TEM)

The brain tissues were prefixed in a mixture of 2% paraformaldehyde and 2.5% glutaraldehyde. Samples were then prepared as previously described [34]. Microglia exhibit distinct ultrastructural features. Their nuclei are irregular or bean-shaped, with dense heterochromatin clumps along the membrane, giving a darker appearance compared to other glial cells. The cytoplasm is electron-dense and contains abundant lysosomes, phagosomes, and lipid droplets, reflecting their phagocytic function, while rough endoplasmic reticulum and Golgi apparatus are less prominent. Excitatory synapses exhibit asymmetric postsynaptic densities with prominent electron density. The image was detected by FEI Talos F200S G2 TEM and STEM (RRID:SCR_019906) operated at 200 kV, 60,000 ╳ magnification.

### Fluorescent Ca^2+^ indicators for imaging cell function

Primary microglia were plated on fibronectin-coated glass-bottom dishes (MatTek Corporation). After 24 h, cells were loaded with 4 μM Fluo-4 AM and 3 μM Fura-red AM (AAT Bioquest, 20,552 and 21,048, China) in the presence of Pluronic Acid F-127 (Merck, P2443, USA) at 37 °C for 30 min. After washing, cells were resuspended in Ca^2^⁺ Ringer's solution for 10 min. Time-lapse images were acquired at 37 °C with 5.2% CO₂ using a Leica SP8 LIGHTNING confocal microscope. Images were analyzed in Fiji to calculate average intensities in green and red channels for each ROI. The Fluo-4/Fura-Red ratio was determined 5 min after ATP induction.

### Calpain activity assay

To measure the calpain activity of primary microglial cells, cells were seeded in a 12-well plate and the medium was changed to a serum-free medium. Cells were treated with 4 µg/µL AAT for 24 h and subjected to a Calpain-Glo™ Protease Assay (Promega, G8501, Wisconsin, USA). The assay was performed according to the manufacturer’s protocol.

### Statistical analysis

All statistical analyses were performed using GraphPad Prism (RRID:SCR_002798). Data are presented as mean ± standard error of the mean (SEM). Statistical comparisons between two independent groups were evaluated using an unpaired, two-tailed Student’s *t*-test. For comparisons involving three or more experimental groups, a one-way or two-way ANOVA was performed, followed by Tukey’s post hoc test for all pairwise comparisons to account for multiple testing. Notably, for electrophysiological data, a nested ANOVA was employed to account for the hierarchical structure of the data, with individual cells nested within their respective animals to avoid pseudoreplication. Statistical significance was predefined as *p* < 0.05.

## Supplementary Information

Supplementary Material 1.

## Data Availability

The raw and processed RNA-sequencing data generated in this study have been deposited in the China National Center for Bioinformation/Beijing Institute of Genomics, Chinese Academy of Sciences (GSA: CRA048855) that are publicly accessible at https://ngdc.cncb.ac.cn/gsa/s/Ew20X8gd. All other data supporting the findings of this study are available from the corresponding author upon reasonable request.

## Abbreviations

α-syn: α-Synuclein
AAT: Alpha-1-antitrypsin
Arg-1: Arginase-1
BBB: Blood–brain barrier
cAMP: Cyclic adenosine 3′,5′-monophosphate
CB2R (*Cnr2*): Cannabinoid receptor 2
CNS: Central nervous system
Co-IP: Co-immunoprecipitation
D1-MSNs: Dopamine receptor type 1-expressing medium spiny neurons
D2-MSNs: Dopamine receptor type 2-expressing medium spiny neurons
DAMP: Danger-associated molecular pattern
EPM: Elevated plus maze
FCT: Fear conditioning test
GAD1: Glutamate decarboxylase 1
GFAP: Glial fibrillary acidic protein
Gsk-3β: Glycogen synthase kinase 3 beta
GO: Gene ontology
ICV: Intra-cerebroventricular injection
IL-1β: Interleukin-1β (*Il1b*)
IL-6 (*Il6*): Interleukin-6
IL-10 (*Il10*): Interleukin-10
IL-18 (*Il18*): Interleukin-18
IFN-γ (*Ifng*): Interferon-γ
IF: Immunofluorescence
iNOS (*Inos*): Inducible nitric oxide synthase
Iba1: Ionized calcium binding adaptor molecule-1
KEGG: Kyoto encyclopedia of genes and genomes
mEPSC: Miniature excitatory postsynaptic currents
MSNs: Medium spiny neurons
NAc: Nucleus accumbens
N-Gsk-3β: N-terminal glycogen synthase kinase 3 beta
NLRP3: NLR family pyrin domain containing 3
NOR: Novel object recognition
PCR: Polymerase chain reaction
PCA: Principal component analysis
PD: Parkinson’s disease
PPI: Protein–protein interaction
pSTAT3: Phosphorylated signal transducer and activator of transcription 3
P^ser9^-Gsk-3β: Phospho-serine 9-glycogen synthase kinase 3 beta
sEPSC: Spontaneous excitatory postsynaptic current
STAT3: Signal transducer and activator of transcription 3
SPR: Surface plasmon resonance
TEM: Transmission electron microscopy
TNF-α (*Tnfa*): Tumor necrosis factor-alpha
WB: Western blot
Ym1/Chi313 (*Ym1*): Chitinase-3-like protein 3
