# Supplementary material for: α1-Antitrypsin attenuates microglial NLR family pyrin domain containing 3 inflammasome activation via cannabinoid receptor 2 signaling to ameliorate α-synucleinopathy-related behavioral deficits
Source: Mol Biomed. 2026 Sep 20;7:180. doi: 10.1186/s43556-026-00583-5 (PMC13590469; doi:10.1186/s43556-026-00583-5)
Supplement: Supplementary file 1 [file 43556_2026_583_MOESM1_ESM.docx]

Supplementary Information

**α1-Antitrypsin Attenuates Microglial NLR Family Pyrin Domain Containing 3 Inflammasome Activation via Cannabinoid Receptor 2 Signaling to Ameliorate α-synucleinopathy-Related Behavioral Deficits**

Linjuan Feng^a,b,e^**^†^**, Hsuan Lo^c^**^†^**, Weipin Weng^d,e^**^†^**, Jiahao Zheng^d,e^, Yixin Sun^a,d,e^, Wei Lin^b^, Xiaochun Chen^a,e^, Yanping Wang^f^*, Xiaodong Pan^d,e^*

*Corresponding author

E-mail: pxd77316@163.com (Xiaodong Pan); ORCID ID: 0000-0001-9511-6986.

E-mail: 447585020@qq.com (Yanping Wang); ORCID ID: 0009-0005-9947-3821.

**^†^**These authors contributed equally to this work.

**This file includes:**

Materials and methods

Figure S1-S6

Table S1- S3

**Materials and methods**

**Behavioral tests**

All behavioral assessments of motor and non-motor functions were conducted during the animals' active phase (lights-on cycle: 10:00-16:00) under controlled dim red light conditions (15 lux). These behavioral experiments were conducted by examiners blinded to the groups, ensuring unbiased and objective evaluations.The details were described in the supplemental files.

**Fear conditioning test (FCT)**

The apparatus used for the conditioning and context test consisted of a square chamber equipped with an electrifiable grid floor, a sound source, and a calibrated shock generator (Ecgonline, China). Initially, mice were allowed 120 seconds of free exploration within the conditioning chamber (Context A). Subsequently, an 85 dB, 7 kHz tone was played for 30 seconds as a conditioning stimulus (CS), with a 0.5 mA footshock administered as an unconditioned stimulus (US) during the final 2 seconds of the sound presentation. This experimental procedure was repeated for five cycles to establish associative learning patterns and elicit specific behavioral responses for further analysis.

After a 24-hour rest period, the mice were placed in Context A for a 5-minute spontaneous activation to test contextual memory. For the cued memory test, the mice were subsequently transferred to a novel context (Context B) consisting of a 2-minute baseline period and a subsequent 30 seconds of the CS, which was repeated for five cycles. Freezing behavior, defined as the absence of movement for ≥1 second, was assessed during the 2-minute baseline period. All trials and data were automatically recorded and analyzed using JLBehv-FCSM-1 software (Ecgonline, China).

**Y maze test**

The Y-shaped maze consists of three white, opaque plastic arms positioned at a 120° angle from each other, with one arm designated as the novel arm and closed off during the training phase. To test spatial reference memory, mice first underwent a free-moving exploration session in the Y-maze to familiarize themselves with the environment. Afterwards, the mice were removed from the maze. After a 4-hour interval, the mice were reintroduced to the maze, with the previously blocked arm now open, and allowed to explore all three arms freely for 10 minutes. The movements of the mice were recorded and analyzed using JLBehv-YMM software (Ecgonline, China). A valid entry was recorded when the center of the animal's body was positioned on the arm. The spontaneous alternation ratio was calculated using the formula: [Triads/(Total entries − 2)] × 100%.

**Elevated plus maze (EPM) test**

The EPM apparatus consists of two open arms (30 × 5 cm) and two closed arms (30 × 5 × 20 cm) arranged in a plus configuration, elevated 70 cm above the floor. Mice were placed at the center of the maze, facing an open arm, and allowed free exploration for 10 min. Movements were automatically monitored and analyzed using JLBehv-EPMM software (Ecgonline, China). Data of entries into the open arms, distance traveled, and time spent in the open arms were recorded and analyzed.

**Novel object recognition (NOR) test**

Mice were introduced into the center of an opaque plexiglas box (45 × 45 × 45 cm) and allowed to freely explore for 10 min to adapt to the environment on the first day. In the training (T1) phase, two identical objects were placed in opposite quadrants of the arena (NE corner and SW corner). Twenty-four hours after acclimation, mice were placed in the center of the arena, equidistant from the 2 identical objects, and allowed free exploration for 10 min. In the testing (T2), 24 h after training, the familiar object used during T1 was replaced with a novel object. Mice were placed in the center and allowed free exploration for 10 min. During experiment their movements were automatically tracked and analyzed by JLBehv-LAM-4 (Ecgonline, China). Alternatively, the preference index (d3) was calculated by the time spent exploring the novel object divided by the total time (d3 = b/e2*100).

**Gait test**

A runway (40 cm long, 5 cm wide) was lined with a piece of white paper at the bottom. Mice were trained to run down the runway in a straight line one day before the test. The paws of mice were coated with red and black non-toxic paint (forepaws red, hindpaws black) and they were allowed to walk through a small tunnel on the white paper. Stride length (distance between the fore paw and rear paw prints), sway length and stance length (distance between the left forepaw and right rear paw prints) were measured.

**Rearing cylinder test**

Mice were placed in a small transparent cylinder (15.5–30 cm high and 12.7–20 cm wide) to encourage vertical exploration and landing after each rearing. The test was videotaped for a predetermined 5-min period. Measurements included the number of wall-assisted or free-standing rears, forelimb and hindlimb steps, and time spent grooming.

**Primary microglia culture**

Primary microglia were prepared as previously described [1]. After 2 weeks of culture, mixed glial cell cultures were shaken at 260 rpm and 37°C for 30 minutes. Microglial suspensions were collected and seeded onto poly-L-lysine-coated plates. The CB2R agonist JWH 133 (Absin, abs810831) and the CB2R antagonist AM630 (Sigma, SML0327) were dissolved in distilled water containing 1% dimethyl sulfoxide (DMSO) and 1% Tween-80 as a stock. The calpain inhibitor calpeptin (Aladdin, 117591-20-5) was dissolved in DMSO as a stock.Microglial cells were incubated for 24 hours with the following: PBS, fibrillar α-syn (500 nM), JWH133 (25 μM) + α-syn, AAT (4 µg/µL) + AM630 (15 μM), AAT + calpeptin (50 μM) in the presence of α-syn. Cells were collected for immunofluorescence (10³ cells/well), qPCR (10⁵ cells/well), western blot (10⁵ cells/well), calpain activity assay, and Ca²⁺ assay (10³ cells/well).

**Enzyme-linked immunosorbent assay (ELISA)**

The concentrations of IL-1β, IL-18, and cAMP were quantified using specific ELISA kits according to the manufacturers' instructions. For cytokine measurements, primary microglia were seeded in 12-well plates and transitioned to serum-free medium prior to treatment with 4 µg/µL AAT for 24 hours. The resulting supernatants were collected and subjected to IL-1β (Jingmei, JM-02323M1) and IL-18 (Jingmei, JM-02452M1) ELISA assays. For intracellular signaling analysis, cAMP levels in cell culture lysates (100 µL) were determined using a high-sensitivity ELISA kit (sensitivity: 0.61 pmol/mL; linear detection range: 1–243 pmol/mL). Absorbance was measured at the specified wavelengths, and the concentration of each analyte was calculated based on the respective standard curves.

**Immunofluorescence**

Brain sections and cultured cells were prepared as previously detailed [1]. To minimize non-specific binding, samples were incubated in a blocking buffer containing 10% normal donkey serum (Sigma-Aldrich, USA) for 1 h at room temperature. Subsequently, sections were incubated overnight at 4°C with primary antibodies (Supplementary Table S3), followed by appropriate species-matched secondary antibodies; nuclei were counterstained with DAPI for visualization. Confocal microphotographs were captured using a Zeiss LSM 780 Confocal Laser Scanning Microscope (RRID: SCR_020922) with consistent acquisition parameters across groups. Protein co-localization within microglia was quantified using ZEISS ZEN software (RRID: SCR_013672), while integrated fluorescence density was analyzed via Fiji (RRID: SCR_002285). For advanced spatial visualization, three-dimensional (3D) surfaces were reconstructed using Imaris software (Bitplane). Skeletal architecture was extracted using the ‘Skeletonize’ tool and the ‘Analyze Skeleton (2D/3D)’ plugin in Fiji to determine the total number of branch segments, defined as the continuous path between nodes or endpoints. Furthermore, microglial complexity was evaluated by Sholl analysis (Fiji), which quantified the number of process intersections across concentric circles originating from the soma (starting radius: 4 μm; radial increment: 3 μm).

**Fig. S1 a** Representative double immunofluorescence images of Iba1 (red) and CB2R (green) in the NAc of CB2R⁺/⁺ and CB2R⁻/⁻ mice at 2 h post-α-syn injection. The merged images confirm the specific colocalization of CB2R with Iba1⁺ microglia in CB2R⁺/⁺ mice and explicitly demonstrate the complete absence of CB2R expression in the CB2R⁻/⁻ cohort despite the strong α-syn pathological challenge. Scale bar, 20 μm (n = 3–5 mice per group). **b** PCR genotyping of the CB2R mouse model resolved on a 1.5% agarose gel. The wild-type allele (CB2R⁺/⁺) yields a 385 bp amplicon, whereas the knockout allele (CB2R⁻/⁻) yields a 550 bp amplicon. Heterozygous mice (CB2R⁺/⁻) exhibit both the 385 bp and 550 bp bands.

**Fig. S2 a** PCA plots show gene expression (FPKM) levels (PC2) and RNA quality (TIN score, PC1) as a function of sample characteristics. Each point represents an RNA-Seq sample and showed the overall similarity (or difference) in gene expression profiles among CB2+/+ and CB2-/- groups following treatment with PBS and fibrillar α-Syn. **b** Venn diagram illustrating the numbers of overlapping and distinct differentially expressed genes among four groups. Quantification of the total number of expressed genes in RNA-seq across four groups, as depicted by bar plots. **c** The Heatmaps shows the degree of up-regulation (red) and down-regulation (blue) of DEGs in the NAc from four groups. The five subclusters exhibited clustering based on similar differential expression patterns of DEGs. **d** Line graph depicting expression trends of all genes within subcluster 5 across four distinct groups. **e** KEGG enrichment chord diagram showing that the DEGs correspond to the top 10 significantly enriched KEGG terms. **f** Pathway Enrichment Analysis revealed interactions among the top 10 KEGG-enriched pathways, with direct relations highlighted in solid lines. The color bar denotes the degree of *p-*value adjustment, while the circle size represents the number of DEGs participating in each path. PCA, principal component analysis; KEGG, kyoto encyclopedia of genes and genomes.

**Fig. S3 a** Colocalization of AAT with microglia. Representative immunofluorescence images showing Iba1 (red) and AAT (green) in the NAc. The right panel displays a line profile analysis (derived from the white line in the merged image), demonstrating the spatial overlap (black arrows) between Iba1 and AAT fluorescence intensities across the microglial cell body. **b-c** Absence of AAT colocalization with neurons and astrocytes. Representative images showing staining for (b) the neuronal marker βIII-tubulin (red) and (c) the astrocyte marker GFAP (red) alongside AAT (green). Note the lack of AAT signal within these specific cell types, confirming microglial specificity. Scale bars, 20 μm for all panels.

**Fig. S4 a-b** Sholl analysis and skeleton analysis of microglia showed the processes and branches between six groups. (n = 6).

**Fig. S5 a-b** Immunofluorescence dual-labeling and quantitative analysis of NLRP3^+^ areas in primary microglia visualized by Iba1 (red) and NLRP3 (green) expressions. Scale bar:  50 μm, 10 μm (n=6). The data are presented as the mean ± SEM and assessed by two-way ANOVA followed by Tukey's multiple comparisons test. ^#^*p* < 0.05, ^##^*p* < 0.01, ^###^*p* < 0.001, between primary microglia treated with PBS, α-Syn, and α-Syn + AAT; **p* < 0.05, ***p* < 0.01, ****p* < 0.001, compared between CB2^+/+^ and CB2^-/-^ primary microglia with different treatment.

**Fig. S6** **a** Schematic representation of the gait test, including the stride length (blue line), stance length (yellow line) and sway length (red line). **b** The percentage of ipsilateral paw use during a 5-minute cylinder test. The data are presented as the mean ± SEM and assessed by two-way ANOVA followed by Tukey's multiple comparisons test (n=14-15).

**Table S1. The primer sequences used for the genotyping.**

| **Mice** | **Name** | **Genotyping Primer Sequence** |
| --- | --- | --- |
| *Cnr2* | ^†^Mutant (5’-3’) | GGG GAT CGA TCC GTC CTG TAA GTC T |
|  | ^†^Common (5’-3’) | GAC TAG AGC TTT GTA GGT AGG CGG G |
|  | ^†^Wild type (5’-3’) | GGA GTT CAA CCC CAT GAA GGA GTA C |

^†^The three different primers used for the genotyping of CB2^+/+^ and CB2^-/-^mice.

**Table S2. Forward and reverse sequences of the used primers.**

| **Gene** | **GeneBank** | **Forward sequence** | **Reverse sequence** |
| --- | --- | --- | --- |
| Arg | [NM_001136104.1](https://www.ncbi.nlm.nih.gov/nuccore/NM_001136104.1) | AGAGCAGGATAGGATGGCAATGAC | TGGTTGAGAAGAGGTGGACACTG |
| GAPDH | NM_008084 | CAGTGGCAAAGTGGAGATTGTTG | CTCGCTCCTGGAAGATGGTGAT |
| IL-1α | [NM_010554.4](https://www.ncbi.nlm.nih.gov/nuccore/NM_010554.4) | GATTCTGAAGAAGAGACGGCTGAG | TCTGGTAGGTGTAAGGTGCTGATC |
| IL-1β | NM_008361 | ATGCCACCTTTTGACAGTGATG | TGATGTGCTGCTGCGAGATT |
| IL-6 | NM_001314054.1 | CTCCCAACAGACCTGTCTATAC | CCATTGCACAACTCTTTTCTCA |
| IL-10 | [NM_010548.2](https://www.ncbi.nlm.nih.gov/nuccore/NM_010548.2) | GGACAACATACTGCTAACCGACTC | TGGATCATTTCCGATAAGGCTTGG |
| INOS | NM_001313921.1 | ACTCAGCCAAGCCCTCACCTAC | TCCAATCTCTGCCTATCCGTCTCG |
| IFN-γ | [NM_008337.4](https://www.ncbi.nlm.nih.gov/nuccore/NM_008337.4) | GGAGGAACTGGCAAAAGGATGG | CAGGTGTGATTCAATGACGCTTATG |
| Serpina1a | NM_001252569.1 | ATTCAGTGTCCTCTCCAGCC | GAGGGAGTCATTTTCCACCGT |
| Serpina1b | NM_009244.4 | CTTCAACAATGGGGCTGACCT | ATAGACATAGGAACGGCTTCAAA |
| Serpina1c | NM_009245.2 | GCAGCAGCTACAGTCTTACTAGC | GGGTGGTCATTTATGTGTGGG |
| Serpina1d | NM_009246.3 | TAAACAGGCGCAGAAGCGAT | GAGGTCAGCCCCATTGTTGA |
| TNF-α | NM_001278601 | ACTGGCAGAAGAGGCACTCC | GCCACAAGCAGGAATGAGAA |
| Ym1 | NM_009892 | GCAGAAGCTCTCCAGAAGCAAT | ATTGGCCTGTCCTTAGCCCA |

**Table S3. The antibodies used in the IF and WB.**

| **Name** | **Company** | **Cat# &** RRID | **Dilution** |
| --- | --- | --- | --- |
| **Primary antibodies** | | | |
| α-synuclein | Santa Cruz (CA, U.S.A.) | Cat# sc-12767  RRID: AB_628318 | 1:1000 |
| AAT | Abcam (Cambridge, UK) | Cat# ab166610  RRID: | 1:500 |
| βIII-tubulin | Abcam (Cambridge, UK) | Cat# ab52623  RRID: AB_869991 | 1:1000 |
| CB2 (3C7) | Santa Cruz (CA, U.S.A.) | Cat# sc-10076  RRID: AB_2082786 | 1:500 |
| Calpain-1 | ABclonal (CA, U.S.A.) | Cat# A18006  RRID: AB_2861802 | 1:1000 |
| Caspase-1+p10+p12 | Abmart (Shanghai, CHN) | Cat# T510200  RRID: non | 1:1000 |
| GAD1 | Cell Signal Technology  (MA, U.S.A.) | Cat# 41318  RRID: AB_2799198 | 1:500 |
| GAPDH | Abcam (Cambridge, UK) | Cat# ab181602  RRID: AB_2630358 | 1:5000 |
| GFAP | Cell Signal Technology  (MA, U.S.A.) | Cat# 3670S  RRID: AB_561049 | 1:1000 |
| IBA1 | Wako Chemicals (Osaka, Japan) | Cat# 019-19741  RRID: AB_839504 | 1:500 |
| IBA1 | Abcam (Cambridge, UK) | Cat# ab5076  RRID: AB_2224402 | 1:500 |
| IBA1 | Novus (CO, U.S.A.) | Cat# NB100-1028  RRID: AB_521594 | 1:500 |
| N-Gsk3β | Cell Signal Technology  (MA, U.S.A.) | Cat# 12456  RRID: AB_2636978 | 1:1000 |
| NLRP3 | Invitrogen (MA, U.S.A.) | Cat# MA5-23919  RRID: AB_2605972 | 1：500 |
| pGsk3β | Cell Signal Technology  (MA, U.S.A.) | Cat# 5558  RRID: AB_10013750 | 1:1000 |
| Procaspase-1 | Abcam (Cambridge, UK) | Cat# ab179515  RRID: AB_2884954 | 1:1000 |
| **Secondary antibodies** | | | |
| Alexa Fluor 488 (Ms) | Thermo Fisher Scientific (MA, U.S.A.) | Cat# A-21202  RRID: AB_141607 | 1:2000 |
| Alexa Fluor 488 (Rb) | Thermo Fisher Scientific (MA, U.S.A.) | Cat# A-21206  RRID: AB_2535792 | 1:2000 |
| Alexa Fluor 594 (Rb) | Thermo Fisher Scientific (MA, U.S.A.) | Cat# A-21207  RRID: AB_141637 | 1:2000 |
| Alexa Fluor 647 (Goat) | Thermo Fisher Scientific (MA, U.S.A.) | Cat# A-21447  RRID: AB_2535864 | 1:2000 |
| Alexa Fluor 594 (Rb) | Thermo Fisher Scientific (MA, U.S.A.) | Cat# A-11012  RRID: AB_2534079 | 1:2000 |
| Alexa Fluor 594 (Ms) | Thermo Fisher Scientific (MA, U.S.A.) | Cat# A-21203  RRID: AB_141633 | 1:2000 |
| Alexa Fluor 488 (Rat) | Thermo Fisher Scientific (MA, U.S.A.) | Cat# A-21208  RRID: AB_2535794 | 1:2000 |
| Alexa Fluor 594 (Rat) | Thermo Fisher Scientific (MA, U.S.A.) | Cat# A-21209  RRID: AB_2535795 | 1:2000 |
| Alexa Fluor 594 (Goat) | Thermo Fisher Scientific (MA, U.S.A.) | Cat# A-11058  RRID: AB_142540 | 1:2000 |
| Alexa Fluor 488 (Goat) | Thermo Fisher Scientific (MA, U.S.A.) | Cat# A-32814  RRID: AB_2762838 | 1:2000 |
| Mouse HRP | Abcam (Cambridge, UK) | Cat# ab6789  RRID: AB_955439 | 1:5000 |
| Rabbit HRP | Abcam (Cambridge, UK) | Cat# ab6721  RRID: AB_955447 | 1:5000 |
| Rat HRP | Invitrogen (MA, U.S.A.) | Cat# 31470  RRID: AB_228356 | 1:5000 |
| Rat HRP | Vector Laboratories (CA, U.S.A.) | Cat# BA-9400  RRID: AB_2336202 | 1:5000 |

References

1. Feng L., Lo H., Hong Z., Zheng J., Yan Y., Ye Z., et al. Microglial LRRK2-mediated NFATc1 attenuates α-synuclein immunotoxicity in association with CX3CR1-induced migration and the lysosome-initiated degradation. *Glia*. 2023; 71(9) 2266-2284. https://doi.org/10.1002/glia.24422.
